# Health-illness transition processes in children with complex chronic conditions and their parents: a scoping review

**DOI:** 10.1186/s12887-024-04919-4

**Published:** 2024-07-11

**Authors:** David Loura, Ana Margarida Ferreira, Joana Romeiro, Zaida Charepe

**Affiliations:** 1 Local Health Unit of São José, Dona Estefânia Hospital, St. Jacinta Marto, N. 8A, 1150-192 Lisbon, Portugal; 2 Faculty of Health Sciences and Nursing, Catholic University of Portugal, Lisbon, Portugal; 3 Local Health Unit of Arco Ribeirinho, Nossa Senhora Do Rosário Hospital, Setúbal, Portugal; 4 Center for Interdisciplinary Health Research (CIIS), Lisbon, Portugal; 5 Catholic University of Portugal, Postdoc-Fellowship Program in Integral Human Development (IHD), CADOS, Lisbon, Portugal

**Keywords:** Child, Chronic disease, Parents, Transitional care, Healthcare

## Abstract

**Background:**

The prevalence of complex chronic conditions (CCC), which cause serious limitations and require specialized care, is increasing. The diagnosis of a CCC is a health-illness transition for children and their parents, representing a long-term change leading to greater vulnerability. Knowing the characteristics of these transitional processes is important for promoting safe transitions in this population. This scoping review aimed to map the available evidence on health-illness transition processes in children with complex chronic conditions and their parents in the context of healthcare.

**Methods:**

Six databases were searched for studies focusing on children aged 0–21 years with CCC and their parents experiencing health-illness transition processes, particularly concerning adaptation to illness and continuity of care, in the context of healthcare. Studies within this scope carried out between 2013 and 2023 and written in Portuguese or English were identified. The articles were selected using the PRISMA methodology. The data were extracted to an instrument and then presented with a synthesizing approach supporting the interpretation of the results.

**Results:**

Ninety-eight methodologically broad but predominantly qualitative articles were included in this review. Children with CCC have specific needs associated with complex and dynamic health-illness transitions with a multiple influence in their daily lives. Several facilitating factors (p.e. positive communication and a supportive therapeutic relationship with parents and professionals, as well as involvement in a collaborative approach to care), inhibiting factors (p.e. the complexity of the disease and therapeutic regime, as well as the inefficient organization and coordination of teams) and both positive (p.e. well-being and better quality of life) and negative response patterns (p.e. negative feelings about the chronic illness) were identified. Some interventions to support the transitional process also emerged from the literature. Pediatric palliative care is seen as a good practice and an integrative approach for these children and families.

**Conclusion:**

Health professionals play a fundamental role in supporting the transitional process and promoting positive response patterns. More significant investment is needed at the clinical and academic levels regarding production and dissemination of knowledge in this area to ensure the awareness of children with CCC and that their needs are fully enhanced.

**Review registration:**

10.17605/OSF.IO/QRZC8.

**Supplementary Information:**

The online version contains supplementary material available at 10.1186/s12887-024-04919-4.

## Background

In line with the growing role of children in society, healthcare for children and young people has been subject to continuous improvement. It is known that illness at the pediatric age is not considered an expected normative process and significantly impacts the entire family system when it arises [1]. Significant advances in biomedical science and technology, which are responsible for the decline in infant mortality rates, have contributed to a change in the healthcare paradigm toward the treatment of children with pathologies for which there was once no therapeutic strategy [2, 3].

This reality has led to an increase in the number of children with Complex Chronic Conditions (CCC), which are defined as any pathology lasting at least one year, involving one or more organ systems enough to result in severe limitations that require specialized pediatric care and, probably, a period of hospitalization in a tertiary health institution [4]. Recently, a concept analysis conducted on complex health conditions in children also highlighted the dynamic nature of these diseases, the uniqueness of their impact on each child and family and the diminished quality of life often inherent to them [5].

In the literature, associated with the complications that arise from a complex chronic illness (CI), CCC is part of a broader set of pathologies, referred to as LLI and life-threatening illnesses (LTI). It is a concept defined by Together for Short Lives as LLI/LTI for which treatment can be effective or fail, which can result in the death of the child or irreversible or progressive conditions [6]. These circumstances can lead to a high degree of disability for the child, as well as a dependence on technology [7–9] and recurrent hospital stays [10].

These conditions are then further subdivided into five major groups: diseases for which there is a life-threatening risk with available treatment but with some degree of fallibility (group 1); diseases for which premature death is inevitable but with which there may be extended survival with appropriate treatment (group 2); progressive diseases with no available treatment (group 3); irreversible but nonprogressive diseases with a high degree of dysfunctionality and vulnerability (group 4); and finally, diseases that affect the fetus or newborn and have incurable features associated with a very short life expectancy (group 5) [6, 11]. The growing prevalence of these conditions now affects more than three million children worldwide [12], and their diagnosis can be seen as a process of transition [13].

According to Meleis and colleagues [14], the concept of transition is based on long-term changes in health, relationships and the environment, which involves adapting to new roles and situations. This process can result in new ways of thinking and acting and will increase internal resilience when an individual is aware of what is happening. The journey may or may not be linear and can lead to negative feelings in individuals associated with the impact that changes and responses to them have on them, placing them in a state of vulnerability [14].

Regardless of the name given to these illnesses, specialized pediatric care is required given their multidimensionality, reflected by high dependence on technological resources (mechanical ventilation, for example), demanding therapeutic management, parenteral and enteral nutrition, and equipment to compensate for vital functions, such as dialysis systems, urinary catheters and ostomies [15]. Given the complexity of these LLIs, extended hospital stays are the most prevalent for these children and their parents [16], with a significant impact on the increase in mortality and associated health expenditures approaching €3,000 per admission [17, 18].

The literature recommends that individualized support for children with CCC be planned and implemented by a specialized multidisciplinary team as early as possible (ideally at the time of diagnosis), supported by psychosocial mechanisms and resources that allow adequate management of the disease in all contexts [19, 20]. We are referring, for example, to approaches such as pediatric palliative care (PPC), which is emerging as a therapeutic strategy that positively contributes to improving the quality of life of these children and their families, not only at the end of life but also as a right for all children with a serious, disabling, incurable and potentially fatal pathology [21, 22].

The CCC and often inherent functional disability, development deficits and therapeutic complexity have a significant impact on family functioning, with the potential for an overload of care, responsibilities and worries that can diminish quality of life [23]. Factors such as the age of the child and parents, financial issues, uncertainty about the future, stigma and social isolation all contribute to this problem [24, 25]. As significant and binding figures for children, parents are often the main carers in this context and are consequently exposed to an adjustment and training process regarding the illness, which can generate insecurities and uncertainties about how to act [26, 27].

In this context, the philosophy of child- and family-centered care becomes fundamental, as it is expressed through holistic, humanized and individualized practices in which the healthcare team can identify the physical and psychosocial needs of the family, involve them in the care plan and give them increasing responsibility in its implementation [28, 29].

The community also plays a significant role in the progressive management of CCC and in its social representation, monitoring health and disease development, coordinating care and referrals to the appropriate resources and, finally, promoting normalization and inclusion, enabling the child and family to maintain their social roles [30, 31].

Faced with these transitional processes, the intervention of professionals in this field is reflected in the concept of “care transition”, defined as a set of interventions designed to ensure effective and safe coordination and continuity of care for people experiencing changes in their health [32]. The literature states that the inclusion of a case manager allocated to direct care in these cases could be an essential solution to support these transitional processes [33–35].

Considering the evidence already produced in this area, the motivation for this scoping review is the need to synthesize the existing knowledge in the literature about the health-disease transition processes in this population. A significant amount of evidence was found after conducting a preliminary search of the literature, justifying its synthesis and mapping on this subject. At the same time, a search was conducted in the PROSPERO, MEDLINE, Cochrane Database of Systematic Reviews and JBI Evidence Synthesis databases. No scoping reviews were found to be finalized or in progress on the subject. Within the scope of this review, the following main research question was defined: What evidence is available on health-illness transition processes in children with a CCC and their parents in the context of healthcare?

To deepen the analysis, five secondary questions are also proposed:

- What is the nature of health-illness transitions in children with CCC and their parents in the context of healthcare?
- Which conditions facilitate transition for children with CCC and their parents in the context of healthcare?
- Which conditions inhibit transition in children with CCC and their parents in the context of healthcare?
- Which health interventions support the health-illness transition in children with CCC and their parents in the context of healthcare?
- What are the patterns of response associated with transition processes in children with CCC and their parents in the context of healthcare?

## Methods

This scoping review was carried out taking into account the guidelines issued by the Joanna Briggs Institute (JBI) [36], as well as the Preferred Reporting Items for Systematic Reviews and Meta-Analyses extension for Scoping Reviews (PRISMA-ScR) methodology [36]. Library experts agreed on and reviewed the methodological aspects of this scoping review. The involvement of two knowledge users was ensured during the development of the review [37–39].

### Inclusion criteria

Concerning participants, this review considered all studies that included children with a diagnosis of a CCC, an LLI or an LTI, meeting the definition of authors specializing in the field [4, 11]. If there was doubt about the classification, the Directory of Life-Limiting Diseases was used [40]. Children with special health needs (SHN) or who depended on long-term care were also considered participants.

All children from birth to adolescence (0 to 18 years old) were considered as the population, as well as young adults with CI and disabilities up to the age of 21 or in cases where the transition to adulthood has not yet occurred. The option for including young adults from 18 to 21 years old is related to the fact that, in a lot of studies and in the experience of the authors, people with this age are still in the pediatric’ scope of action due to delay in transition to an adult health setting.

All studies involving parents were also identified, regardless of the type of family. Studies involving people over the age of 21 and their families, who were in the context of adult care, were excluded, as were studies involving children with noncomplex CI or an acute pathology requiring health care.

All the studies that focused on the concept of health-disease transition in this population were considered, in line with Meleis' definition [14], even if described under a different name.

Meleis defines "transition" as "(…) a passage or movement from one state, condition or place to another" [41]. This concept can be divided into three dimensions: types (developmental, situational, health-illness and organizational), which describe different situations in which transitional processes occur; patterns (singular/multiple, sequential/simultaneous, related/unrelated), which demonstrate the complexity and plurality of the presentation of these processes; and properties (awareness, involvement, change and difference, period and critical events), which are seen as elements associated with a transition [14, 42]. Transitions can also be influenced by facilitating or hindering personal, community and social factors [14].

According to this theory, the intervention of professionals is therefore based on a comprehensive understanding of the transition to develop congruent 'therapies' in the face of the unique experience of the person and their family, promoting a healthy response to the transition [14]. Therefore, two moments are of specific interest to this review: the physical and psychological adaptation of the child and parents resulting from the diagnosis of a CCC, hospitalization or exacerbation of the underlying condition; their discharge; and the necessary coordination and integration of care. The studies should identify areas for optimization, contributions, or implications for the clinical practice of health professionals, especially nurses.

Studies that exclusively identified transition processes other than health-illness – situational, organizational and/or developmental (i.e., the transition process from healthcare in a pediatric environment to an adult environment) – were excluded.

The context considered in the studies included in this review was the healthcare environment to which the population above resorts, whether at a primary, secondary or tertiary level, and regardless of clinical specialty or economic sector. No sociodemographic restrictions will be applied. Studies other than those with a clear focus on providing direct care were excluded.

Regarding the type of evidence, primary studies with qualitative, quantitative, or mixed methodologies; literature reviews (narrative or systematic); published theses; and case studies were considered. Letters to the editor, opinion articles, editorials, columns, commentaries, and book reviews were excluded.

### Search strategy

The research strategy was developed through several phases per the JBI guidelines.

The first phase corresponded to the initial exploratory search conducted in various databases to identify the literature attributes most pertinent to answering the review question. This research helped to develop a comprehensive search strategy, namely, by identifying the most frequent keywords in natural or indexed language and the words in the titles and abstracts of the most relevant articles.

The second phase corresponded to the search and involved identifying articles that met the inclusion criteria. The final version of the search expression (See Additional file 1), based on a table of search terms (See Additional file 2) with the natural language in English and Portuguese and the indexed terms, was applied to the following databases: CINAHL Complete®, MEDLINE Complete® and Psychology and Behavioral Sciences Collection®, belonging to the EBSCOHost platform; on the Cochrane Library® platform, including the Cochrane Database of Systematic Reviews® and Cochrane Central Register of Clinical Trials® databases; and on the Open Access Scientific Repositories in Portugal (RCAAP) platform, as well as on the OpenAire portal. Opting for these databases was a decision based on the pertinence, scope and coverage of the articles found in the first phase of the search in each database, which made the authors choose the databases with better capability to provide the most extensive and comprehensive search.

The final search expression in natural language is defined in Table 1. Due to their length, the other specific expressions broken down by database are available, as mentioned above, in Additional File 2. The search expression for the OpenAire portal has been simplified, as the system does not support advanced search expressions.

**Table 1:** Search expression in nonindexed language

Search expression in nonindexed language – English	(child* OR parent* OR caregiv*) AND (“chronic disease” OR “chronic illness” OR “chronic condition” OR “complex chronic condition” OR “life-limiting condition” OR life-threatening condition” OR “long-term conditions” OR “long-term care” OR “complex care” OR “special health needs) AND (transition* OR continuity OR adapt* OR diagnos*) AND (nurs* OR pediatric* OR “pediatric palliative care”)

The search was performed in April 2023 at Psychology and Behavioral Sciences Collection ® and RCAAP (in Portuguese). Other search expressions are available in Additional File 1

Articles published in any scientific publication were considered, regardless of their nature, provided that they were written in Portuguese or English and published in the last ten years (between 2013 and 2023). The restriction in chronological terms was due to the need to make scientific evidence compatible with the current reality of care, which has evolved exponentially around children with CCC in recent years. In addition, transferring the most up-to-date knowledge to clinical practice was also an important consideration when selecting evidence for the review.

### Selection of evidence

After the search, the studies’ records were extracted and uploaded to a bibliographic management support system (Zotero), where the process of identifying the articles was carried out, checking the records’ information and eliminating duplicates. The materials were then uploaded to bibliographic review support software (Rayyan), where the eligibility assessment and selection of articles took place [43].

Article screening was conducted by two reviewers, starting by analyzing the titles and abstracts, where the authors looked for compliance with inclusion criteria regarding population, concept and context. The assessment of the complete text was done by overlapping results and conclusions of each study with the defined review questions. A final decision in the face of disagreements was reached by consensus between the reviewers, and there was no need to call by a third reviewer. The reasons for excluding articles after complete text analysis are summarized in a table (see Additional file 3). At the end of the process, the various stages were represented in a PRISMA-ScR flow diagram [36, 44, 45].

### Data extraction

After the search, information was extracted (in April 2023) through an instrument including data on the authors, the study’s country of origin, its type, objective, sample, methodology and results, aligned with evidence recommendations [39, 46]. The results were then reorganized and grouped according to their contribution to answering the review questions, making it easier to synthesize the evidence and align it with the outlined objective. This method allowed for a multimodal analysis, by review question and by article, which was also useful to connect results with the underlying theoretical framework. As this was a scoping review, the quality of the studies included was not assessed.

### Data analysis and presentation

**Fig. 1:**
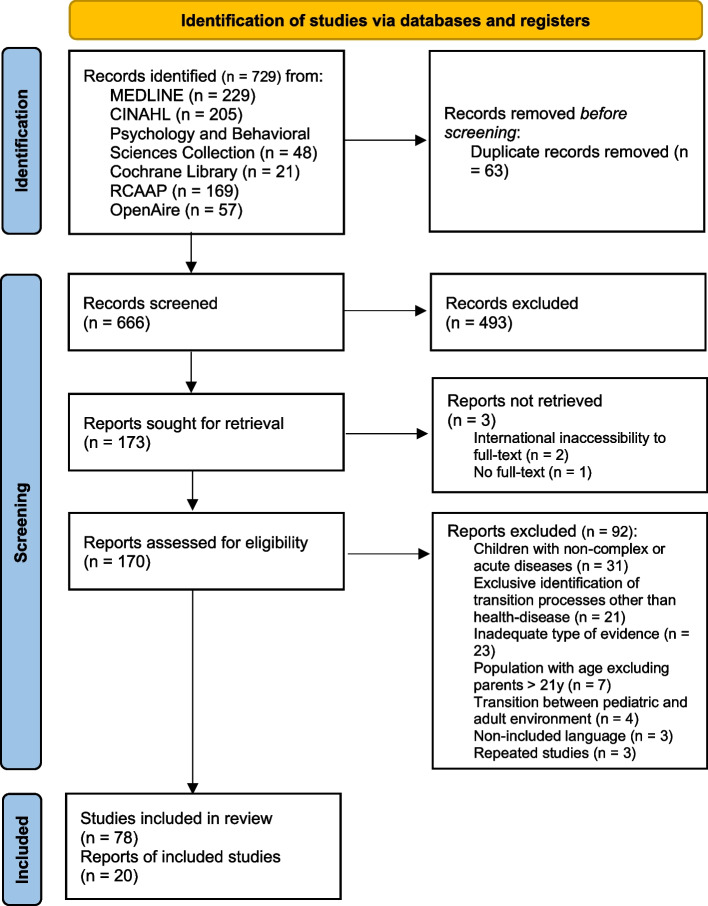
PRISMA-ScR flow diagram for the identification, screening and inclusion of studies in the review

The data from this review are presented in an integrative approach with a schematic view of the content. The search results and selection of the relevant studies are presented in the PRISMA-ScR flowchart in the results’ section (Fig. 1). Analysis of the data was carried out through an inductive methodology, following further categorization of the results by review question. This method allowed for a meaningful exploration of the results.

## Results

### Included studies

The inclusion of studies in the review followed the methodology specified above and was translated into a PRISMA-ScR flow diagram (Fig. 1). After the identification and screening processes, 78 articles were included in the review. The studies’ bibliographic references were checked, and 20 additional articles were included.

### Characteristics of the included studies

The included studies were diverse in their typology, with multidisciplinary authorship in 98 of the included articles, demonstrating the multiprofessional interest in transitional care and, more specifically, in children with CCC.

Approximately 80% of the articles are primary, and most are qualitative, with interviews and questionnaires being the most common techniques used to collect data. One article on concept analysis, a less common methodology, also contributed to this review.

**Fig. 2:**
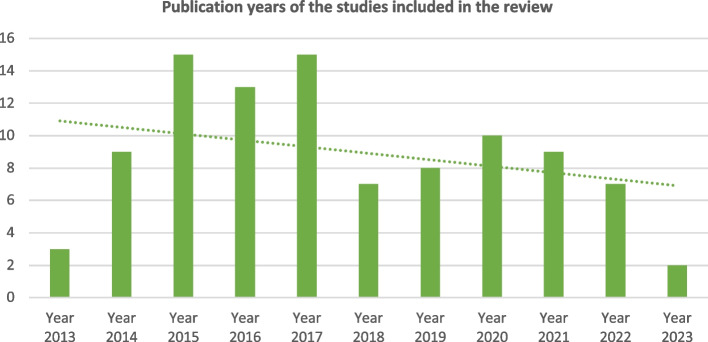
Graph – Dates of publication regarding the studies included in the review

For the years of publication of the articles included, there is considerable asymmetry between the first five years of the sample (2013–2017) and the last six years (2018–2023), as there has been a considerable decrease in the evidence published on this subject over time. The number of publications peaked in 2015 and 2017. The evidence published in the last two years comprises less than 10% of the sample. Figure 2 shows a graph illustrating these trends.

**Fig. 3:**
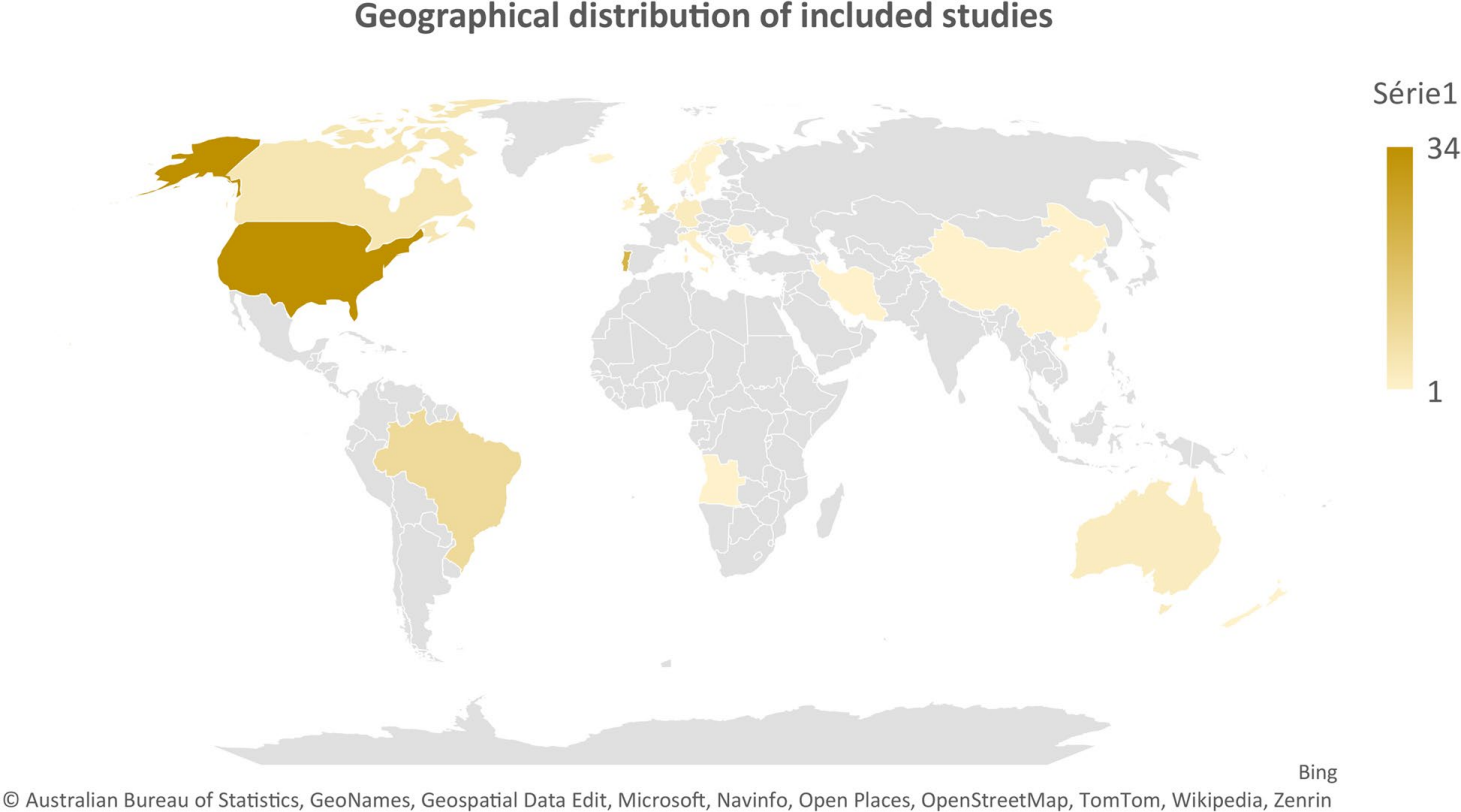
Graph – Geographical distribution of the included studies

Regarding the geographical distribution, illustrated in Fig. 3, the high geographical dispersion of the studies stands out, with the United States of America being the country with the most scientific production on transitional processes in children with CCC and their parents (*n* = 34).

### Main results

The included articles made essential contributions to answering the outlined review questions. To better describe the characteristics of each study, as well as its objectives and results, these data are summarized in a table in the appendix to this article (see Additional file 4).

**Fig. 4:**
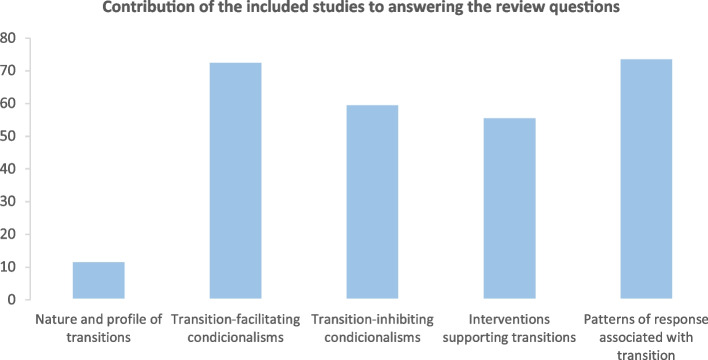
Graph – Contribution of the included studies to answering the review questions

Below, a graph (Fig. 4) shows that most of the literature focuses on the facilitating and inhibiting constraints of the transitional processes of children with CCC and their parents and the response patterns demonstrated by this population. Nevertheless, a significant proportion of the literature on multidisciplinary interventions associated with these transitional processes is beginning to emerge. Given its applicability in the health sciences, this analysis was conducted through the theoretical lens of Afaf Meleis [14, 47].

To systematically organize the results, we will describe the main conclusions associated with the review questions outlined.

#### Nature of existing health-disease transitions

In this section, articles focused on defining who these children are and what their transitional needs are. In the literature, children with CCC are given numerous names: children with medical complexity (CMC), children with SHN, children with complex health needs, children with LLI or LTI, and children with palliative needs, among others [48, 49].

These transitions are generally considered to be health-related diseases. Nevertheless, some evidence suggests that they are also situational and organizational, with a simultaneous and interrelated pattern. The specificity of these transitional processes is linked to several factors, such as the complexity of the pathologies, their multisystemic involvement, the high impact on the quality of life of the child and the family [48–51], high functional dependence and socioemotional needs [48, 50, 52–54].

Care management seems to have the most significant weight in defining these transitions, given the need for frequent and coordinated multiprofessional health surveillance, with wide-ranging therapeutic strategies to control symptoms, from polymedication to the use of technological devices to support vital functions [48, 49, 51, 52]. Although there are few tools for assessing the needs of this population, which often causes them to be recognized too late, the evidence shows that the prevalence of these diseases is increasing worldwide [48, 55–57].

#### Transition-facilitating conditionalisms

**Fig. 5:**
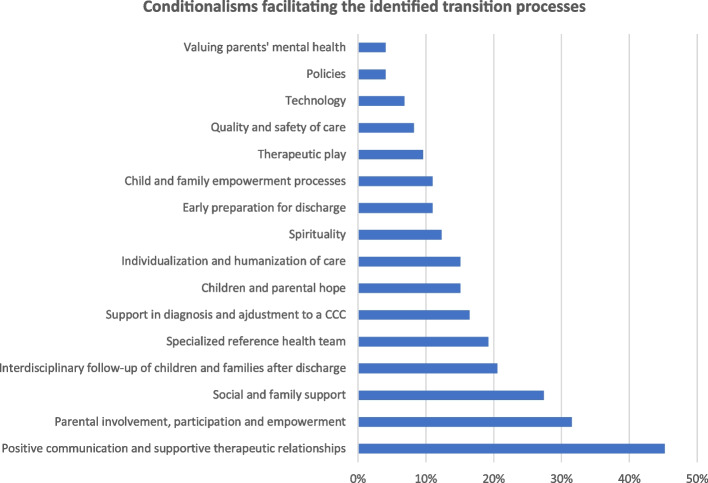
Graph – Conditionalisms facilitating the identified transition processes

Children and parents identify many constraints facilitating health-disease transitions, influencing the adoption of positive response patterns. A summary diagram of the presence of these constraints in the literature analyzed is shown in Fig. 5.

Throughout the care process, the adoption of a positive and supportive relationship and communication style between the child, parents and professionals is the main facilitator of adjustment to the illness identified by the evidence, mainly when it includes explicit communication, a sense of trust in professionals and an affectionate and available tone in the therapeutic relationship [51, 53, 54, 58–87]. This attitude is also conveyed in the individualization and humanization of care, which is manifested in respect to the choice of the child and family in the care process [65, 75, 78, 79, 81, 83, 88–92]. In this context, using recreational resources is particularly important [64, 65, 71, 74, 78, 93, 94].

Providing care in partnership with parents and children is another facilitator, advocating for collaborative approaches that integrate them as active elements in the planning and implementation of care [53, 58, 59, 62, 63, 70, 71, 75, 79–85, 90, 91, 93, 95–105]. In this regard, training and learning knowledge and skills about the disease and the therapeutic regime are essential [58, 79, 90, 92, 98, 103, 106, 107], the quality of which is enhanced by the existence of reference professionals or case managers [52, 58, 75, 81, 83, 88, 91, 92, 95, 97, 99, 107–109].

From a more personal perspective, hope, optimism, spirituality and trust in children's potential for independence are factors that help children overcome these experiences [53, 63, 64, 69–71, 74–76, 79, 82, 87, 93, 96, 105, 110–113]. At the same time, valuing parents' mental health is seen as a resource to alleviate the potential burden that can fall on them [50, 65, 112]. Social and family support also appears to be positive, particularly if there are siblings in the family and if there are opportunities to join formal or informal mutual support networks [50, 53, 54, 59, 74, 76, 82, 85, 87, 96, 100, 103, 105, 106, 112, 114–118].

The evidence also identifies the importance of valuing the discharge process, highlighting the importance of knowing housing conditions before leaving the hospital and planning for follow-up in the community. [54, 65, 73, 75, 85, 90, 98, 99, 101, 105, 107, 108, 114, 117, 119, 119–123]. Clinical support by telephone or continuity of care at home are also valued approaches [65, 73, 75, 99, 101, 114, 122, 123].

From a more systemic perspective, there is evidence that the perception of safety and quality of care impacts how children and parents experience these transitional processes [61, 67, 90, 91, 114, 124]. Similarly, policies to increase access to support services, as well as adequate socioeconomic conditions and the use of technological devices, are strategies associated with positive outcomes [52, 59, 60, 65, 74, 116, 123, 125].

#### Transition-inhibiting conditionalisms

Several factors that can negatively influence the successful completion of a transition stand out.

The existence of several prejudices associated with the necessary healthcare and the negative progression of the disease can make it difficult for a child with a CCC and their parents to accept the condition, aggravating uncertainty about the future [53, 55, 60, 64, 65, 67, 89, 93, 96, 106, 126–128].

The complexity of the disease and the therapeutic regimen have been identified as the main factors hindering these transition processes, as these pathologies involve uncontrolled symptoms, functional limitations, a demanding therapeutic regimen and recurrent clinical follow-ups. [52, 59, 64, 67, 77, 91, 93, 96, 98, 104, 106, 108, 112, 117, 122, 126, 127, 129]. Therefore, dysfunctional therapeutic relationships can amplify this negative impact [54, 59, 91, 92, 101].

Some professionals' resistance to caring for these children and their families can hinder the training process and thus lead to inadequate preparation for discharge [54, 59, 60, 64, 77, 101, 104, 110, 130]. This phenomenon can also occur when there is a high turnover of professionals, especially nurses, reducing the consistency of support for decision-making and training [59, 81, 88, 110].

At discharge, erratic communication and coordination between professionals are also barriers to continuity of care [59, 60, 64, 64, 73, 73, 74, 81, 88, 93, 97, 101, 108, 119, 127, 128, 131]. This process may be hampered by the inadequacy of care facilities in the community to accommodate a child with these needs [59, 74, 128].

On a personal level, there are also several important factors. For parents, the process of adapting to their child's CI leads to a decrease in socialization and family support [59, 65, 79, 82, 87, 96, 111, 114, 123, 127]. The decline in sleep quality and consequent parental stress therefore seems inevitable, complicated by the caregiver's limited access to respite resources [50, 54, 65, 67, 72, 96, 128, 132–134].

For children, their limitations contrast with peer pressure to carry out activities typical for their age group [53, 64, 67, 79, 89, 93, 96, 126, 127]. School absenteeism has also been reported as a consequence of the need for frequent clinical follow-up, sometimes leading to overprotective parental practices in the face of the risk of complications [93, 107, 127].

From a social perspective, the literature describes stigma, underrecognition of fathers as caregivers and barriers to parents’ professional life as existing problems [106, 128]. These obstacles make it difficult for parents to maintain their social roles and can lead to economic difficulties, given the often high cost of their children's treatment [54, 60, 65, 69, 87, 96, 103, 106, 108, 111, 112, 116, 123, 127, 130]. The lack of public policies tailored to the complexity of children with CI is also an obstacle to this process [123, 129].

#### Health interventions that support transition

The evidence suggests that these interventions are multidisciplinary and involve multiple professionals (doctors, nurses, physiotherapists, psychologists, social workers, and spiritual counselors, among others) [106, 113, 130, 133, 135–137]. The implementation of palliative care as an integrative approach for children with CCC and their families is seen as good practice [51, 55, 56, 91, 102, 106, 124, 125, 138]. The assignment of a reference professional, mainly identified as a nurse who coordinates the child's clinical situation using case management methodology, is also recommended [48, 88, 108, 136, 139].

Recognizing the needs of these children as early as possible before intervention is a procedure described in the evidence. Therefore, the emergence of validation studies of diagnostic instruments associated with this end, in terms of the child and the caregivers, is seen as an added value [57, 68, 128]. Improving these tools is fundamental to the process of advanced care planning, which should focus on priorities defined in a shared and individualized way [53, 57, 60, 68, 137, 140–142].

In this context, a trend toward involving children in decision-making has emerged from the evidence. Although children may not yet have the necessary discernment to take full responsibility for decisions regarding their health, some authors report the need to respect their perspective, avoiding empowering parents exclusively and promoting their participation and involvement in training (when possible) [75, 89, 119].

Regarding direct care, the prioritization of training and empowerment as a means to develop self-management and self-efficacy is seen as a safe transition-promoting intervention [50, 53, 54, 58, 71, 73–75, 80, 81, 92, 93, 97, 98, 104, 108, 115, 131, 135], along with strength-based and hopeful communication [53, 70, 75, 76, 84, 89, 140].

The evidence also focuses on the discharge process, highlighting the crucial availability of community resources allowing for continuity of care. Therefore, coordination between hospital and community teams to define the discharge strategy early on is one of the mentioned interventions [49, 51, 60, 63, 80, 86, 95, 106, 120, 123, 125, 135, 138, 139, 142, 143]. In this regard, technology-based solutions, particularly telehealth care, are relevant [73, 95, 135].

In addition, the implementation of support mechanisms for caregiver performance, such as programs promoting respite, as well as participation in mutual help groups with professional support, can contribute to the development of hope and self-efficacy, fostering positive feelings and enhancing a safe transition [53, 54, 63, 91, 100].

#### Patterns of response associated with transition processes

From the evidence, more positive response patterns are associated with these transitions, as opposed to less favorable ones. This duality is summarized in Tables 2 and 3, associating the response patterns with their magnitude in the literature analyzed.

**Table 2:** Positive patterns of response regarding the health-disease transition by number (N) and percentage (%)

Positive patterns of response	N	%
Positive feelings about chronic disease management [52–54, 62, 64, 66, 67, 69, 75, 78, 79, 83, 84, 87, 88, 91, 94, 96, 103, 111, 113, 117, 121, 126, 127, 134, 142, 144]	28	38%
Well-being and quality of life [49, 53, 61, 69, 71, 80, 82, 83, 91, 95, 104, 106, 108, 110, 113, 115, 137, 139, 142]	19	26%
Optimized self-efficacy in managing the therapeutic regimen [52, 53, 71, 74, 83, 92, 93, 105, 108, 113, 115, 118, 119, 121, 135]	15	20%
Reduction of complications associated with complex chronic disease [83, 86, 93, 95, 106, 119, 122, 135, 138]	9	12%
Effective parental role [58, 63, 80, 85, 102, 104, 105, 117, 126]	9	12%
Improved family functioning [62, 63, 69, 84, 85, 103, 117, 134]	8	11%
Child’s symptomatic control [69, 85, 86, 106, 124, 136]	6	8%
Balancing the caregiver role with personal and professional life [66, 67, 95, 96, 103, 135]	6	8%
Acceptance of complex chronic illness [74, 87, 96, 117]	4	5%

**Table 3:** Negative patterns of response regarding the health-disease transition by number (N) and percentage (%)

Negative patterns of response	N	%
Negative feelings about the chronic illness [54, 64, 67, 72, 76, 79, 88, 96, 98, 103–105, 107, 108, 111, 112, 116, 117, 126, 133, 141, 143–145]	24	32%
Social isolation and reduced quality of life [67, 72, 77, 82, 87, 93, 96, 107, 112, 126]	10	14%
Decreased quality of care for the child [98, 105, 108, 118, 131, 132, 134, 145]	8	11%
Decreased family functioning [64, 87, 111, 112, 118, 127]	6	8%
Care overload [67, 79, 87, 123, 133, 134]	6	8%
Difficulty accepting the disease [93, 96, 111]	3	4%
Increased need for hospitalization and clinical follow-up [108]	1	1%

## Discussion

The main objective of this scoping review was to map the available evidence on health-illness transition processes in children with CCC and their parents in the context of healthcare, taking into account the facilitating and inhibiting factors of this transition, as well as the nature and profile of the transition/population, the response patterns associated with the transition and interventions that support the transition [14, 47]. In this sense, the results met the outlined objective.

The studies were diverse and provided a solid answer to the outlined review questions. There was a predominance of primary studies, particularly qualitative (*N* = 44), which may be because these transitional processes involve the experiences of the child and the parents, which are assessed in greater depth using these methodologies. On the other hand, the number of literature reviews is small, accounting for less than 10% of the sample. This review seeks to identify the existing evidence and contribute to the synthesis of available knowledge.

For the number of existing publications, the evidence seems to reflect a growing pattern between 2013 and 2017, which may be associated with the global *boom* in PPC. Increased awareness of this issue has also been evident, with advanced scientific and associative activity on PPC. An example of this is the *Hats On for Children's Palliative Care* (#HatsOn4CPC) event, led in 2013 by the International Children's Palliative Care Network, which promoted global involvement and recognition of PPC through the dissemination of photos wearing different hats. In contrast, there appears to be a less pronounced focus on publications between 2018 and 2023, which may be justified by the emergence of other epidemiological priorities, namely, COVID-19, from 2020 onward.

Regarding the content analysis of the included studies, categorization using Meleis's transition theory helped to organize the contribution of the evidence to answering the review questions [14]. There are many studies related to facilitators, barriers and patterns of response by children and parents to the transitional processes associated with CCC. At the same time, studies integrating structured interventions to support these transitions are beginning to appear in the evidence.

The evidence identifies these transitions as originating from mechanisms predominantly related to health-disease processes, to which organizational and situational aspects are added. This illustrates the multidimensionality of these processes’ clinical and psychosocial situations, justifying a differentiated intervention that values the SHN, the specificity of the care and the clinical complexity present in these cases [6].

All the constraints included in Meleis' theoretical approach, except for meanings, appear in the evidence as facilitators and inhibitors of these transitional processes. This tendency may be related to the unique way in which each child and parent respond to transition processes, which seems to have a multifactorial cause. It appears that preparation and knowledge can facilitate and hinder these transitional processes in similar proportions to social and economic factors, which is in line with the complexity of these processes. However, regarding cultural beliefs, attitudes and community resources, a more significant potential for facilitating transitional processes is identified, which is naturally associated with the considerable impact of these factors on experiencing CCC. Personal factors related to the meaning of these conditions are presented exclusively as inhibitors, in line with some of the articles that illustrate prejudices about these children.

Concerning the nature of the interventions identified to respond to transitional processes, an essential discrepancy is visible, since 8 out of 10 articles discuss therapeutic intervention approaches (*N* = 49) rather than preventive interventions, which were mentioned in less than 40% of the studies. This could be explained by the global gaps in caring for these children [146, 147], exhibiting a focus on immediate needs and relegating preventive aspects to second place. In line with the recommendations of the American Academy of Pediatrics for preventive health care in children, this aspect raises concerns related to continuity of care and valuing children's health, which is achieved through a preventive approach separate from the purely therapeutic sphere [148].

The response patterns shown by the evidence reflect that negative ones are more prevalent in process indicators than in outcome indicators. In this context, more than 50% of the articles showed positive response patterns in both areas. This difference may be related to the stage of acceptance of the disease, where understandably, more negative indicators arise at the time of diagnosis and "shock" at the time of diagnosis. Thus, negative response patterns at this stage justify the need for more targeted intervention at this level at an early stage, preventing these less healthy responses from persisting.

Recognizing the needs of these children was one of the points raised as being important in defining the profile of the transitional processes experienced by these children and families. This is an existing discussion insofar as the management of these situations is primarily the responsibility of health professionals. Several studies in the literature, one of which was identified in this review, attempt to classify the needs of these children using a measuring instrument, such as the Pediatric Palliative Screening Scale (PaPaS) [149], or categorization, such as the Directory of Life-Limiting Diseases, drawn up by Hain and Devins [40], or complexity, using the Pediatric Medical Complexity Algorithm proposed by Simon and colleagues [150].

PPC has also emerged as one of the most appropriate therapeutic solutions for these children, assumed as a right of the child with an LLI or LTI [151]. This is perennially corroborated by the evidence, which identifies them as an integrated strategy that contributes to improving the quality of life of these children, who are the primary users of this care [11, 12]. The multidimensionality of this intervention, centered on the needs expressed by the child and family, allows for the clarification of the prognosis and the definition of a plan that is truly aligned with their priorities, maximizing functionality and the right to health care that safeguards their dignity [152–155].

The assignment of a reference professional, often mentioned as a nurse, is also highlighted, in which the evidence identifies significant benefits, such as high satisfaction with care, shorter hospital stays and increased therapeutic limitations at the end of life [156, 157]. The implementation of successful projects in this area regarding complex chronic diseases in pediatrics is also described, with the role of case managers generating gains compatible with those found in this review [33, 34, 158, 159]. However, there is also evidence of the inherent difficulties associated with the turnover of professionals in the inpatient setting, their potential overload and the high emotional demand [160].

The review also mentions the communication approach as the main facilitator of the transitional process. In the pediatric context, adequate communication is combined with the challenge of making it appropriate for the child's age group and sociorelational maturity. The evidence indicates that a relationship with the child, through active listening, encouraging positive affirmations, fostering trust and setting realistic priorities about the future, promotes hope in the child [161–163].

In palliative care, the complexity of situations often requires a systematized approach to communicating bad news, with the SPIKES approach being recommended as the most appropriate [164]. While barriers to communication in this area have also been identified, multidisciplinary work, a focus on prognosis and taking siblings into account when defining and implementing the communication strategy are trends identified by the latest evidence for optimizing care in this area [165–167].

Parental empowerment is also one of the central interventions associated with positive response patterns. These results align with evidence pointing to the importance of empowering parents in the short, medium, and long term in their adaptation to their child’s CCC [168–170]. Recent studies have shown the need to innovate in this training, mainly through simulation [171, 172]. The need to improve parental self-management assessment of children's CI is also a parent-related concern expressed in the evidence, as an article that recently validated a self-management assessment tool for parents of children with CI (*S-scan-parental self-management support*) [173].

The evidence also shows a tendency for the child's opinion to be considered in the therapeutic processes to which they are subjected. This trend is corroborated by the growing body of evidence on the subject, which argues that each child is unique and that their participation in decision-making is a crucial determinant of their involvement in the management of CCC [29, 174, 175].

The transition to home has the potential to both facilitate and hinder the transitional process. In this sense, the evidence recommends a humanized discharge process with early planning, which promotes continuity of care beyond the hospital setting [15, 176]. Intervention in this process, mainly through an educational approach that values the parents' experience, is associated with health gains for integrating care [177, 178].

Considering that this process can generate family readjustment, it is important to conduct multidisciplinary social and family assessments before discharge [179]. The literature highlights hope during this assessment as significant through the genogram and ecomap of hope, seeking to perceive internal and external resources that can contribute to the success of the therapeutic plan [180]. Considering that the potential impact of the disease on caregivers and children is also pertinent, the use of support dynamics to prevent stress and overload should be a priority for health professionals [181, 182]. Thus, the possibility of respite care for caregivers is fundamental and should be encouraged worldwide [183].

Although CCC and palliative care are often associated with the end of life, the results of the review did not particularly highlight these aspects. However, the evidence shows that the dynamics of grief are important in regard to transitional processes in this area, and interventions should be implemented not only for the grief of parents and family but also for the grief of professionals, avoiding complications inherent in prolonged grief [184–187]. The involvement of bereaved parents in the development of bereavement intervention programs in PPC has been reported to stimulate the development of hope and the rediscovery of meaning for their child's death [188].

From a sociological perspective, the importance of developing policies to support the transitions that children and families experience in this context was also mentioned in the results. This perspective is corroborated by scientific evidence, which reports that the fragility of the health system in providing care for these children can generate disorganization and deconstruction of the bond with health professionals [189, 190]. To this end, the World Health Organization's recommendations urge all countries to develop more comprehensive PPC services to improve access to this care worldwide [22].

The results of this review are summarized in Additional File 5, which is organized using the *Pager Framework*. This approach is designed to maximize the quality of the analysis of articles in scoping reviews, which advocates identifying patterns in the evidence analyzed and then classifying the results into advances, gaps, evidence for clinical practice and recommendations for research [191].

Concerning the limitations of this review, it should be noted that, as a secondary study, the results may not reflect all the evidence available on this subject. The exclusion of articles referring to noncomplex CI and the transition from a pediatric to an adult care setting limited the results inherent in these transitional processes. The purpose of this review was to identify existing studies, and for this reason, the quality of the included studies was not assessed. As this is not the correct type of study to achieve a complete transfer of evidence to clinical practice, although it can guide areas of epistemological development in this area, this work does not replace future systematic reviews of the literature, particularly qualitative, diagnostic accuracy, and prevalence studies, which could make a more solid contribution to transferring knowledge to the provision of care.

## Conclusion

By carrying out this scoping review, it was possible to map scientific evidence about the health-illness transition processes in children with CCC and their parents in healthcare.

Due to the complexity that a CCC requires from health professionals, parents and society, this topic has emerged as essential because of the human rights of life, dignity, freedom and security, which are intrinsic to all human beings, and the Sustainable Development Goals, which aim to achieve the well-being of all populations by the year 2030 [192].

Children and families in this context are often presented with the need to face the complexity of a CCC and its therapeutic regime, which can be worsened by economic difficulties, stress, overload of care and unemployment. Although experiencing the impact of a CCC is a difficult situation, parental involvement and participation in care, as well as therapeutic play and the search for community-based resources to support transition between the hospital and home, are strategies that can be led by the family in order to facilitate the transition process, always in partnership with the health professionals.

Health professionals play a fundamental role in minimizing negative constraints on the health-disease transition and positively maximizing them by considering the response patterns associated with the disease and the uniqueness of each child and family. Designing holistic and integrative care for these families, through a positive therapeutic relationship with a reinforcement of education and empowerment processes, coordination between the hospital and the community and the implementation of pediatric palliative care, can promote increased self-management skills and acceptance of the CCC, which can contribute to a positive and safe health-illness transition.

With respect to research, it is important to continue investigating PPC and children with CCC, highlighting the contributions that literature reviews can have in transferring knowledge to care practice contexts. More significant investment is needed at the clinical and academic levels in the production and dissemination of knowledge in this area to increase awareness of children with CCC and their needs.

The low amount or absence of public policies targeting support to children with CCC and their families is a factor that can exacerbate complications when living with this conditions. Policy-makers should therefore invest in recognizing these people’ needs and, linking them to scientific and reliable evidence from research, offer children and family-friendly policies aiming to mitigate some of the barriers they face, particularly at the community, economic and social level. Everyone has the right to live beside the disease, even if it is life-limiting or threatening.

### Supplementary Information

Supplementary Material 1.

Supplementary Material 2.

Supplementary Material 3.

Supplementary Material 4.

Supplementary Material 5.

## Data Availability

All data generated or analyzed during this study are included in this published article (and its supplementary information files).

## Abbreviations

CCC: Complex Chronic Condition
CI: Chronic Illness
CIC: Chronically Ill Children
CMC: Children with Medical Complexity
DM1: Diabetes Mellitus Type 1
ED: Emergency Department
JBI: Joanna Briggs Institute
LLI: Life-Limiting Illness
LTI: Life-threatening illness
PaPaS: Pediatric Palliative Screening Scale
PICU: Pediatric Intensive Care Unit
PPC: Pediatric Palliative Care
PRISMA-ScR: Preferred Reporting Items for Systematic Reviews and Meta-Analyses extension for Scoping Reviews
SHN: Special Health Needs

## References

[1] Cushman G, Shih S, Reed B (2020). Parent and family functioning in pediatric inflammatory bowel disease. Child Basel Switz.

[2] Muñiz GG. Soins palliatifs en Pédiatrie: perspectives et tendances. In: Astudillo W, Astigarraga I, Salinas A, Mendinueta C, Navajas A, D’Souza C, et al., editors. Médecine palliative chez les enfants et adolescents. 1st ed. Saint-Sébastien: Palliatif Sans Frontières; 2021. p. 27–38.

[3] Vasconcelos Da Silva L, Correia Reis B, Da Costa Rodrigues Lima F, Apolinario Alencar V. Profile of children with special health needs (CSHN) in home care. Comun Em Ciênc Saúde. 2023;33(04). Available from: https://revistaccs.escs.edu.br/index.php/comunicacaoemcienciasdasaude/article/view/1318

[4] Feudtner C, Christakis DA, Connell FA (2000). Pediatric deaths attributable to complex chronic conditions: a population-based study of Washington State, 1980–1997. Pediatrics.

[5] Azar R, Doucet S, Horsman AR, Charlton P, Luke A, Nagel DA (2020). A concept analysis of children with complex health conditions: implications for research and practice. BMC Pediatr.

[6] Together for Short Lives (2018). A Guide to Children’s Palliative Care.

[7] Amirnovin R, Aghamohammadi S, Riley C, Woo MS, Del Castillo S (2018). Analysis of a pediatric home mechanical ventilator population. Respir Care.

[8] Bellieni CV. A new holistic-evolutive approach to pediatric palliative care. Cham: Springer International Publishing; 2022. Available from: 10.1007/978-3-030-96256-2.

[9] Cruz S, Fernandes C, Magalhães B (2023). A scoping review of mobile apps for use with palliative patients in the context of home care. Int J Med Inf.

[10] Glassman P (2017). Interventions focusing on children with special health care needs. Dent Clin North Am.

[11] Benini F, Papadatou D, Bernadá M, Craig F, De Zen L, Downing J (2022). International Standards for Pediatric Palliative Care: From IMPaCCT to GO-PPaCS. J Pain Symptom Manage.

[12] Worldwide Hospice and Palliative Care Alliance. Global Atlas of Palliative Care. 2nd Edition. London: WHPCA; 2020. Available from: http://www.thewhpca.org/resources/global-atlas-on-end-of-life-care

[13] Somanadhan S, Brinkley A, Larkin PJ (2022). Living through liminality? Situating the transitional experience of parents of children with mucopolysaccharidoses. Scand J Caring Sci.

[14] Meleis AI, Sawyer LM, Im EO, Hilfinger Messias DK, Schumacher K (2000). Experiencing transitions: an emerging middle-range theory. Adv Nurs Sci.

[15] Horiguchi L, Carvalho LL, Umeda E, Borba M (2022). Harmonious performance of a multidisciplinary health team: humanized disinternment. Rev Bioét.

[16] Araújo YB de, Santos SR dos, Neves NT de AT, Cardoso ÉL da S, Nascimento JA (2020). Predictive model of hospitalization for children and adolescents with chronic disease. Rev Bras Enferm.

[17] Bell J, Lingam R, Wakefield CE, Fardell JE, Zeltzer J, Hu N (2020). Prevalence, hospital admissions and costs of child chronic conditions: a population-based study. J Paediatr Child Health.

[18] Lacerda AF, Oliveira G, Cancelinha C, Lopes S (2019). Hospital inpatient use in Mainland portugal by children with complex chronic conditions (2011–2015). Acta Médica Port.

[19] Charepe Z. Children and young people with chronic or disabling illnesses. In: Ramos AL, Barbieri-Figueiredo M do C, editors. Enfermagem em saúde da criança e do jovem. 1st Edition. Lisbon: Lidel; 2020. p. 231–7.

[20] Walker M, Nicolardi D, Christopoulos T, Ross T (2023). Hospital, hospice, or home: a scoping review of the importance of place in pediatric palliative care. Pall Supp Care.

[21] International Children’s Palliative Care Network. The ICPCN Charter of Rights for life limited and life threatened children. ICPCN; 2008. Available from: https://www.icpcn.org/icpcn-charter/.

[22] World Health Organization. Integrating palliative care and symptom relief into paediatrics: a WHO guide for health-care planners, implementers and managers. Geneva: World Health Organization; 2018. Available from: https://apps.who.int/iris/handle/10665/274561

[23] Rozensztrauch A, Kołtuniuk A (2022). The quality of life of children with epilepsy and the impact of the disease on the family functioning. Int J Environ Res Public Health.

[24] Aoun SM, Stegmann R, Deleuil R, Momber S, Cuddeford L, Phillips MB (2022). “It Is a whole different life from the life i used to live”: assessing parents’ support needs in paediatric palliative care. Children.

[25] Freitas FC. Needs and quality of life of parents who are primary caregivers and children accompanied by an in-hospital support team in pediatric palliative care [Master Thesis]. Coimbra: Nursing School of Coimbra; 2021. Available from: http://web.esenfc.pt/?url=NBXd5kl6

[26] Nap-van der Vlist MM, van der Wal RC, Grosfeld E, van de Putte EM, Dalmeijer GW, Grootenhuis MA (2021). Parent-child dyadic coping and quality of life in chronically diseased children. Front Psychol.

[27] Nayeri ND, Roddehghan Z, Mahmoodi F, Mahmoodi P (2021). Being parent of a child with congenital heart disease, what does it mean? A qualitative research. BMC Psychol.

[28] Seniwati T, Rustina Y, Nurhaeni N, Wanda D (2023). Patient and family-centered care for children: A concept analysis. Belitung Nurs J.

[29] Silveira A, Huppes G, Soster F, Bueno T, Bartsch L, Maurício M (2021). Each child is a child: singularity of children with special health needs. J Nurs Health.

[30] Bem C, Small N (2020). An ecological framework for improving child and adolescent health. Arch Dis Child.

[31] Jorge AM, Carrondo EM, Lopes FMT (2016). Pediatric palliative home care focused on the family: contributions toward a salutogenic orientation. Egitania Sci.

[32] Dusek B, Pearce N, Harripaul A, Lloyd M (2015). Care transitions: a systematic review of best practices. J Nurs Care Qual.

[33] Bourque M, DeFilippis D, Adkins L (2021). Creating a new model of care by integrating case management nurses in a children’s hospital. Prof Case Manag.

[34] Mesa-Melgarejo L, Carreño Moreno S, Chaparro-Diaz L, Quintero González LA, Garcia-Quintero D, Carrillo-Algarra AJ (2022). Effectiveness of a case management model for people with multimorbidity: mixed methods study. J Adv Nurs.

[35] Tavares P, Santos Silva R, Magalhães B (2022). Factores determinantes en la transición a cuidados paliativos: perspectiva de enfermeros expertos. OncoNews.

[36] Tricco AC, Lillie E, Zarin W, O’Brien KK, Colquhoun H, Levac D (2018). PRISMA Extension for Scoping Reviews (PRISMA-ScR): checklist and explanation. Ann Intern Med.

[37] Aromataris E, Munn Z. JBI Manual for Evidence Synthesis. JBI; 2020. Available from: https://jbi-global-wiki.refined.site/space/MANUAL/4687342/Chapter+11%3A+Scoping+reviews.

[38] Jull JE, Davidson L, Dungan R, Nguyen T, Woodward KP, Graham ID (2019). A review and synthesis of frameworks for engagement in health research to identify concepts of knowledge user engagement. BMC Med Res Methodol.

[39] Pollock D, Peters MDJ, Khalil H, McInerney P, Alexander L, Tricco AC (2023). Recommendations for the extraction, analysis, and presentation of results in scoping reviews. JBI Evid Synth.

[40] Hain RD, Devins M. Directory of Life-Limiting conditions. 2011. Available from: https://www.togetherforshortlives.org.uk/resource/directory-life-limiting-conditions/. 10.1186/1472-684X-12-43 PMC4029745 24330676

[41] Chick N, Meleis AI. Transitions: a nursing concern. In: Chinn P, editor. Nursing research methodology. Boulder, CO: Aspen Publication; 1986. p. 237–57. Available from: https://repository.upenn.edu/nrs/9.

[42] Schumacher KL, Meleis Al (1994). Transitions: a central concept in nursing. Image J Nurs Sch.

[43] Ouzzani M, Hammady H, Fedorowicz Z, Elmagarmid A (2016). Rayyan—a web and mobile app for systematic reviews. Syst Rev.

[44] Purssell E, McCrae N. How to perform a systematic literature review: a guide for healthcare researchers, practitioners and students. Cham: Springer International Publishing; 2020. Available from: 10.1007/978-3-030-49672-2.

[45] Page MJ, McKenzie JE, Bossuyt PM, Boutron I, Hoffmann TC, Mulrow CD, The PRISMA (2020). statement: an updated guideline for reporting systematic reviews. BMJ.

[46] Peters M, Godfrey C, Mclnerney P, Munn Z, Tricco A, Khalil H. Chapter 11: Scoping reviews. In: JBI Manual for Evidence Synthesis. JBI; 2020. Available from: https://jbi-global-wiki.refined.site/space/MANUAL/4687342/Chapter+11%3A+Scoping+reviews.

[47] Lindmark U, Bülow PH, Mårtensson J, Rönning H, Ahlstrand I, A.D.U.L.T. Research Group (2019). The use of the concept of transition in different disciplines within health and social welfare: An integrative literature review. Nurs Open.

[48] Rogers J, Reed MP, Blaine K, Manning H (2021). Children with medical complexity: a concept analysis. Nurs Forum (Auckl).

[49] Galligan MM, Bamat TW, Hogan AK, Piccione J (2018). The pediatric aerodigestive center as a tertiary care-based medical home: a proposed model. Curr Probl Pediatr Adolesc Health Care.

[50] Palma E, Deatrick JA, Hobbie WL, Ogle SK, Kobayashi K, Maldonado L (2015). Maternal caregiving demands for adolescent and young adult survivors of pediatric brain tumors. Oncol Nurs Forum.

[51] Deming RS, Mazzola E, MacDonald J, Manning S, Beight L, Currie ER (2022). Care intensity and palliative care in chronically critically Ill infants. J Pain Symptom Manage.

[52] Caicedo C (2016). Children with special health care needs: child health and functioning outcomes and health care service use. J Pediatr Health Care.

[53] Hill DL, Miller V, Walter JK, Carroll KW, Morrison WE, Munson DA (2014). Regoaling: a conceptual model of how parents of children with serious illness change medical care goals. BMC Palliat Care.

[54] Lopes VC do R. Information/Training Needed for Parents Caring for Children with Special Health Needs at Home [Master Thesis]. [Lisbon]: Portuguese Catholic University; 2016. Available from: http://hdl.handle.net/10400.14/19731.

[55] Connor SR, Downing J, Marston J (2017). Estimating the global need for palliative care for children: a cross-sectional analysis. J Pain Symptom Manage.

[56] Fraser LK, Gibson-Smith D, Jarvis S, Norman P, Parslow RC (2021). Estimating the current and future prevalence of life-limiting conditions in children in England. Palliat Med.

[57] Palaré MJ, Tavares F, Machado MDC (2023). Validation of the European Portuguese version of a pediatric palliative needs assessment tool: the pediatric palliative screening scale. Acta Médica Port.

[58] Alves JMN de O, Amendoeira JJP, Charepe ZB (2017). The Parental Care Partnership in the View of Parents of Children with Special Health Needs. Rev Gaucha Enferm.

[59] Alves S, Fontaine A (2016). Parents’ emotional needs and difficulties in the face of pediatric palliative illness. Proc - Qual Health Res.

[60] Antolick MM, Looman WS, Cady RG, Kubiatowicz K (2020). Identifying and communicating postdischarge goals for hospitalized children with medical complexity: a process improvement pilot in a specialty pediatric setting. J Pediatr Health Care.

[61] Barata ASF das N. Young children’s satisfaction with diabetes consultation: impact on their quality of life [Master Thesis]. Viseu: Viseu Higher School of Health; 2016. Available from: http://hdl.handle.net/10400.19/3172

[62] Bennett H. Parents’ experience of advance care planning: a grounded theory of re-constructing meaning through advance care planning. [Tese de Doutoramento]. University of Southampton; 2020. Available from: https://eprints.soton.ac.uk/452354/

[63] Carvalho MIMP. Promoting self-care in families with children and adolescents with chronic illness [Master Thesis]. Lisbon: Nursing School of Lisbon; 2014. Available from: http://hdl.handle.net/10400.26/16213

[64] Ciobanu E, Preston N (2021). Hearing the voices of children diagnosed with a life-threatening or life-limiting illness and their parents’ accounts in a palliative care setting: A qualitative study. Palliat Med.

[65] Collins A, Hennessy-Anderson N, Hosking S, Hynson J, Remedios C, Thomas K (2016). Lived experiences of parents caring for a child with a life-limiting condition in Australia: a qualitative study. Palliat Med.

[66] Di Riso D, Cambrisi E, Bertini S, Miscioscia M (2020). Associations between pretend play, psychological functioning and coping strategies in pediatric chronic diseases: a cross-illness study. Int J Environ Res Public Health.

[67] Dunbar H, Carter B, Brown J (2020). ‘Place bonding’ in children’s hospice care: a qualitative study. BMJ Support Palliat Care.

[68] Fernandes A, Batalha L, Perdigão A, Campos C de, Nascimento L, Jacob E. Cultural validation of the adolescent pediatric pain tool (APPT) in Portuguese children with cancer. Rev Enferm Referência. 2015;Série IV(4):99–105.

[69] Fernandes RVR. Quality of life and anxiety in the context of pediatric oncology: the role of rituals, cohesion and hope [Master Thesis]. Lisbon: Faculty of Psychology, University of Lisbon; 2018. Available from: http://hdl.handle.net/10451/37933

[70] Fonseca RJ de S. The influence of therapeutic letters on the hope of parents of children with chronic illness [Master Thesis]. Lisbon: Portuguese Catholic University; 2015. Available from: http://hdl.handle.net/10400.14/19730

[71] Govender M, Bowen RC, German ML, Bulaj G, Bruggers CS (2015). Clinical and neurobiological perspectives of empowering pediatric cancer patients using videogames. Games Health J.

[72] Hamner T, Latzman RD, Latzman NE, Elkin TD, Majumdar S (2015). Quality of life among pediatric patients with cancer: contributions of time since diagnosis and parental chronic stress. Pediatr Blood Cancer.

[73] Looman WS, Antolick M, Cady RG, Lunos SA, Garwick AE, Finkelstein SM (2015). Effects of a telehealth care coordination intervention on perceptions of health care by caregivers of children with medical complexity: a randomized controlled trial. J Pediatr Health Care Off Publ Natl Assoc Pediatr Nurse Assoc Pract.

[74] Lopes DID. Nursing interventions in child and family adherence to the type I diabetes therapeutic regimen [Bachelor Thesis]. Oporto: Fernando Pessoa University; 2019. Available from: http://hdl.handle.net/10284/8688

[75] Lotz JD, Daxer M, Jox RJ, Borasio GD, Führer M (2017). “Hope for the best, prepare for the worst”: a qualitative interview study on parents’ needs and fears in pediatric advance care planning. Palliat Med.

[76] Magão MTG. Hope in action: the experience of hope in parents of children with a chronic illness [Doctoral Thesis]. [Lisbon]: University of Lisbon; 2017. Available from: http://hdl.handle.net/10451/40069.

[77] Mavis AM, Ertl A, Chapman S, Cassidy LD, Lerret SM (2015). Vulnerability and chronic illness management in pediatric kidney and liver transplant recipients. Prog Transplant Aliso Viejo Calif.

[78] Melo ASM. The Power of Encounter: The Impact of Hospital Clowns’ intervention on children and adolescents undergoing chemotherapy treatment [Doctoral Thesis]. Braga: Minho University; 2018. Available from: https://hdl.handle.net/1822/55010

[79] Monteiro AIB. Parental experience of pediatric cancer: the couple’s relationship and the impact on parenting [Master Thesis]. Lisbon: Faculty of Psychology, University of Lisbon; 2019. Available from: http://hdl.handle.net/10451/41642

[80] Mororó DD de S, Menezes RMP de, Queiroz AAR de, Silva CJ de A, Pereira WC (2020). Nurse as an integrator in healthcare management of children with chronic condition. Rev Bras Enferm.

[81] Murphy S, Ehritz C (2021). Clinical nurse specialist practice strategies for children with medical complexity. Clin Nurse Spec J Adv Nurs Pract.

[82] Oakley S, Dunbar H, de Vries K (2022). Parent-led strategies supporting personal well-being when caring for a child with a life-limiting condition: a scoping review. J Child Health Care.

[83] Schütze D, Engler F, Ploeger C, Ulrich L, Hach M, Seipp H (2022). Specialised outpatient paediatric palliative care team-parent collaboration: narrative interviews with parents. BMJ Support Palliat Care.

[84] Svavarsdottir EK, Kamban SW, Konradsdottir E, Sigurdardottir AO (2020). The impact of family strengths oriented therapeutic conversations on parents of children with a new chronic illness diagnosis. J Fam Nurs.

[85] Verberne L, Kars M, Schouten-van Meeteren A, Bosman D, Colenbrander D, Grootenhuis M (2017). Aims and tasks in parental caregiving for children receiving palliative care at home: a qualitative study. Eur J Pediatr.

[86] Wells S, O’Neill M, Rogers J, Blaine K, Hoffman A, McBride S (2017). Nursing-led home visits post-hospitalization for children with medical complexity. J Pediatr Nurs.

[87] Wightman A, Taylor Zimmerman C, Neul S, Lepere K, Cedars K, Opel D (2019). Caregiver experience in pediatric dialysis. Pediatrics.

[88] Baird J, Rehm RS, Hinds PS, Baggott C, Davies B (2016). Do you know my child? Continuity of Nursing care in the pediatric intensive care unit. Nurs Res.

[89] Kars MC, Grypdonck MHF, De Bock LC, Van Delden JJM (2015). The parents’ ability to attend to the “voice of their child” with incurable cancer during the palliative phase. Health Psychol.

[90] Leyenaar JK, O’Brien ER, Leslie LK, Lindenauer PK, Mangione-Smith RM (2017). Families’ priorities regarding hospital-to-home transitions for children with medical complexity. Pediatrics.

[91] Ling J, Payne S, Connaire K, McCarron M (2016). Parental decision-making on utilisation of out-of-home respite in children’s palliative care: findings of qualitative case study research - a proposed new model. Child Care Health Dev.

[92] Nightingale R, Friedl S, Swallow V (2015). Parents’ learning needs and preferences when sharing management of their child’s long-term/chronic condition: a systematic review. Patient Educ Couns.

[93] Aguiar GB, Machado MED, da Silva LF, Burla de Aguiar RC, Christoffel MM (2021). Children with type 1 diabetes mellitus: the experience of disease. Rev Esc Enferm USP.

[94] Rindstedt C (2014). Children’s strategies to handle cancer: a video ethnography of imaginal coping. Child Care Health Dev.

[95] Cady RG, Erickson M, Lunos S, Finkelstein SM, Looman W, Celebreeze M (2015). Meeting the needs of children with medical complexity using a telehealth advanced practice registered nurse care coordination model. Matern Child Health J.

[96] Cipolletta S, Marchesin V, Benini F (2015). Family functioning as a constituent aspect of a child’s chronic illness. J Pediatr Nurs.

[97] Fernandez HGC, Moreira MCN, Gomes R (2019). Making decisions on health care for children / adolescents with complex chronic conditions: a review of the literature. Ciênc Saúde Coletiva.

[98] Lerret SM, Weiss ME, Stendahl GL, Chapman S, Menendez J, Williams L (2015). Pediatric solid organ transplant recipients: transition to home and chronic illness care. Pediatr Transplant.

[99] Looman WS, Presler E, Erickson MM, Garwick AW, Cady RG, Kelly AM (2013). Care coordination for children with complex special health care needs: the value of the advanced practice nurse’s enhanced scope of knowledge and practice. J Pediatr Health Care.

[100] Mooney-Doyle K, Deatrick JA, Ulrich CM, Meghani SH, Feudtner C (2018). Parenting in childhood life-threatening illness: a mixed-methods study. J Palliat Med.

[101] Nageswaran S, Radulovic A, Anania A (2014). Transitions to and from the acute inpatient care setting for children with life-threatening illness. Pediatr Clin North Am.

[102] Noyes J, Hastings RP, Lewis M, Hain R, Bennett V, Hobson L (2013). Planning ahead with children with life-limiting conditions and their families: development, implementation and evaluation of ‘My Choices’. BMC Palliat Care.

[103] Pereira MCR. The reconciliation of the spheres of professional, family and personal life: The case of caregivers of children with chronic poetry [Master Thesis]. [Setúbal]: polytechnic institute of Setúbal; 2018. Available from: http://hdl.handle.net/10400.26/25504.

[104] Sousa PCMM de. Parental exercise during the child’s hospitalization: therapeutic nursing intentions in relation to the care partnership [Doctoral Thesis]. Oporto: Portuguese Catholic University; 2014. Available from: http://hdl.handle.net/10400.14/13972

[105] Yazdani N, Chartrand J, Stacey D (2022). Exploring parental decision making for a child with a life-limiting condition: an interpretive description study. J Hosp Palliat Nurs.

[106] Andrade MA. Pediatric palliative care: from hospital to home [Master Thesis]. Lisboa: Faculty of Medicine, University of Lisbon; 2020. Available from: http://hdl.handle.net/10451/47018

[107] da Silva Pimentel RR, Targa T, da Cruz Scardoelli MG (2017). From diagnosis to the unknown: perceptions of parents of children and adolescents with diabetes mellitus. J Nurs UFPE.

[108] Branowicki PA, Vessey JA, Temple KLJ, Lulloff AJ (2016). Building Bridges from hospital to home: understanding the transition experience for the newly diagnosed pediatric oncology patient. J Pediatr Oncol Nurs.

[109] Nolte-Buchholtz S, Zernikow B, Wager J (2018). Pediatric patients receiving specialized palliative home care according to german law: a prospective multicenter cohort study. Children.

[110] Lafrenaye S, Dumas M, Gosselin É, Duhamel A, Bourgault P (2021). Parents living with a child afflicted by a life-limiting medical condition: typology of their narrative identity. Qual Res Med Healthc.

[111] Leite Colesante MF, Gomes IP, de Morais JD, Collet N (2015). Impact on mothers’ lives of caring for children with chronic illnesses. Rev Enferm UERJ.

[112] Pelentsov LJ, O’Shaughnessy P “Kevin,” Laws TA, Esterman AJ. What are the supportive care needs of parents caring for a child diagnosed with ectodermal dysplasia: a rare genetic disorder? Int J Child Health Hum Dev. 2013;7(1):23–9.

[113] Weaver MS, Wratchford D (2017). Spirituality in adolescent patients. Ann Palliat Med.

[114] Desai PP, Rivera AT, Backes EM (2016). Latino caregiver coping with children’s chronic health conditions: an integrative literature review. J Pediatr Health Care Off Publ Natl Assoc Pediatr Nurse Assoc Pract.

[115] Lerret SM, Johnson NL, Haglund KA (2017). Parents’ perspectives on caring for children after solid organ transplant. J Spec Pediatr Nurs.

[116] Macaulay GC, Boucher SE, Yogarajah A, Galland BC, Wheeler BJ (2020). Sleep and night-time caregiving in parents of children and adolescents with type 1 diabetes mellitus – a qualitative study. Behav Sleep Med.

[117] Silva MSM. The experience of parents in relation to adolescent children with type 1 diabetes mellitus [Master Thesis]. Viseu: Viseu Higher School of Health; 2014. Available from: http://hdl.handle.net/10400.19/2538

[118] Zhang Y, Wei M, Zhang Y, Shen N (2014). Chinese family management of chronic childhood conditions: a cluster analysis. J Spec Pediatr Nurs.

[119] Berry JG, Blaine K, Rogers J, McBride S, Schor E, Birmingham J (2014). A framework of pediatric hospital discharge care informed by legislation, research, and practice. JAMA Pediatr.

[120] Carter B, Bray L, Sanders C, van Miert C, Hunt A, Moore A (2016). “Knowing the places of care”: how nurses facilitate transition of children with complex health care needs from hospital to home. Compr Child Adolesc Nurs.

[121] Góes FGB, Cabral IE (2017). Hospital discharge in children with special health care needs and its different dimensions. Rev Enferm UERJ.

[122] McKissick HD, Cady RG, Looman WS, Finkelstein SM (2017). The impact of telehealth and care coordination on the number and type of clinical visits for children with medical complexity. J Pediatr Health Care Off Publ Natl Assoc Pediatr Nurse Assoc Pract.

[123] Seear M, Kapur A, Wensley D, Morrison K, Behroozi A (2016). The quality of life of home-ventilated children and their primary caregivers plus the associated social and economic burdens: a prospective study. Arch Dis Child.

[124] Côté AJ, Payot A, Gaucher N (2019). Palliative care in the pediatric emergency department: findings from a qualitative study. Ann Emerg Med.

[125] Verberne LM, Schouten-van Meeteren AY, Bosman DK, Colenbrander DA, Jagt CT, Grootenhuis MA (2017). Parental experiences with a paediatric palliative care team: a qualitative study. Palliat Med.

[126] Ferreira AF. Parents’ Experience in Pediatric Palliative Care [Bachelor Thesis]. Oporto: Fernando Pessoa University; 2021. Available from: http://hdl.handle.net/10284/10476

[127] Silva ACC da. Biopsychosocial Risk Profile of Adolescents with Chronic Illness [Master Thesis]. [Coimbra]: Faculty of Medicine, University of Coimbra; 2018. Available from: http://hdl.handle.net/10316/81875.

[128] Stochitoiu IA, Vadeboncoeur C (2020). Adaptation and feasibility of the interRAI family carer needs assessment in a pediatric setting. Health Serv Insights.

[129] Kuo DZ, Goudie A, Cohen E, Houtrow A, Agrawal R, Carle AC (2014). Inequities in health care needs for children with medical complexity. Health Aff (Millwood).

[130] Sebastião AM. Physiotherapy intervention in infantile cerebral palsy in Luanda [Master Thesis]. Lisbon: Polytechnic Institute of Lisbon; 2016. Available from: http://hdl.handle.net/10400.21/8039

[131] Mellblom AV, Korsvold L, Finset A, Loge J, Ruud E, Lie HC (2015). Providing information about late effects during routine follow-up consultations between pediatric oncologists and adolescent survivors: a video-based, observational study. J Adolesc Young Adult Oncol.

[132] Gonçalves R, Martins C, Borges A, Madureira N, Cancelinha C (2022). Home mechanical ventilation in children: impact on the sleep quality of caregivers. Port J Pediatr.

[133] Martins CFH. Home ventilation in pediatric complex chronic illness: what impact on caregivers’ quality of life and sleep? [Master Thesis]. [Coimbra]: Coimbra University; 2019. Available from: https://hdl.handle.net/10316/89851.

[134] Romano AEMG. Family resilience in the context of pediatric chronic illness: impact of family caregiver distress and post-traumatic growth on parental caregiver coping responses [Master Thesis]. Lisboa: ISPA; 2021. Available from: http://hdl.handle.net/10400.12/8068

[135] Foster CC, Jacob-Files E, Arthur KC, Hillman SA, Edwards TC, Mangione-Smith R (2017). Provider perspectives of high-quality pediatric hospital-to-home transitions for children and youth with chronic disease. Hosp Pediatr.

[136] Gien J, Kinsella J, Grenolds A, Abman SH, Baker CD, Thrasher J (2017). Retrospective analysis of an interdisciplinary ventilator care program intervention on survival of infants with ventilator-dependent bronchopulmonary dysplasia. Am J Perinatol.

[137] Sterni LM, Collaco JM, Baker CD, Carroll JL, Sharma GD, Brozek JL (2016). An official American thoracic society clinical practice guideline: pediatric chronic home invasive ventilation. Am J Respir Crit Care Med.

[138] Carvalho AJL, Ferreira HM, Borges EF, Borges Junior LH, de Paula ALT, Hattori WT (2019). Analyses of the effectiveness of a Brazilian pediatric home care service: a preliminary study. BMC Health Serv Res.

[139] Young M (2023). Inside the Indiana complex care coordination collaborative. Hosp Case Manag.

[140] Brunetta J, Fahner J, Legemaat M, van den Bergh E, Krommenhoek K, Prinsze K (2022). Age-appropriate advance care planning in children diagnosed with a life-limiting condition: a systematic review. Children.

[141] Ramalho ELR,  da Nóbrega VM, de Mororó DD S, Pinto JTJM, Cabral CHK, Collet N (2022). Nurse’s Performance in the Hospital Discharge Process of Children with Chronic Disease. Rev Gaucha Enferm.

[142] Ulisses LDO, Bispo TAS, Caldas AB, Camargo CL, Oliveira MMC, da Silva EA (2021). Nursing actions for the dehospitalization of children under mechanical ventilation. Acta Paul Enferm.

[143] Hirschfeld RS, Barone S, Johnson E, Boss RD (2019). Pediatric chronic critical illness: gaps in inpatient intrateam communication. Pediatr Crit Care Med.

[144] Muscara F, McCarthy MC, Rayner M, Nicholson JM, Dimovski A, McMillan L (2020). Effect of a videoconference-based online group intervention for traumatic stress in parents of children with life-threatening illness: a randomized clinical trial. JAMA Netw Open.

[145] Nikfarid L, Rassouli M, Borimnejad L, Alavimajd H (2015). Chronic sorrow in mothers of children with cancer. J Pediatr Oncol Nurs Off J Assoc Pediatr Oncol Nurses.

[146] Capelas M, Afonso T, Durão S, Teves C. Care activity of palliative care teams/services. In: Observatório Português dos Cuidados Paliativos: Relatório Outono 2019. Lisbon: Universidade Católica Editora; 2019. p. 44–80. Available from: http://hdl.handle.net/10400.14/30230

[147] Caruso Brown AE, Howard SC, Baker JN, Ribeiro RC, Lam CG (2014). Reported availability and gaps of pediatric palliative care in low- and middle-income countries: a systematic review of published data. J Palliat Med.

[148] Hackell M, Almendarez YM, Berhane AM, Cantrell PE, Kafer LM, Committee on Practice and Ambulatory Medicine (2023). 2023 Recommendations for Preventive Pediatric Health Care. Pediatrics.

[149] Bergstraesser E, Paul M, Rufibach K, Hain RD, Held L (2014). The paediatric palliative screening scale: further validity testing. Palliat Med.

[150] Simon TD, Cawthon ML, Stanford S, Popalisky J, Lyons D, Woodcox P (2014). Pediatric medical complexity algorithm: a new method to stratify children by medical complexity. Pediatrics.

[151] International Children’s Palliative Care Network. Declaration of Cape Town. ICPCN; 2009. Available from: https://www.icpcn.org/wp-content/uploads/2015/06/THE-ICPCN-DECLARATION-OF-CAPE-TOWN-2009.pdf.

[152] Adams S, Nicholas D, Mahant S, Weiser N, Kanani R, Boydell K (2019). Care maps and care plans for children with medical complexity. Child Care Health Dev.

[153] Hawley PH (2014). The bow tie model of 21st century palliative care. J Pain Symptom Manage.

[154] Ospedale Pediatrico Bambino Gesù. Charter of rights for incurable children. Ospedale Pediatrico Bambino Gesù; 2018. Available from: https://img.ospedalebambinogesu.it/images/2021/04/23/122341259-611cb5bf-d21d-43d9-ad5e-76f6348c8c07.pdf

[155] Together for Short Lives. Care Planning in Advance. 2023. Available from: https://www.togetherforshortlives.org.uk/get-support/supporting-you/family-resources/care-planning-advance/.

[156] Edwards JD, Jia H, Baird JD (2021). The impact of eligibility for primary attendings and nurses on PICU length of stay. J Crit Care.

[157] Edwards JD, Williams EP, McHale BL, Lucas AR, Malone CT (2023). Parent and provider perspectives on primary continuity intensivists and nurses for long-stay pediatric intensive care unit patients. Ann Am Thorac Soc.

[158] Brenner M, Doyle A, Begley T, Doyle C, Hill K, Murphy M (2021). Enhancing care of children with complex healthcare needs: an improvement project in a community health organisation in Ireland. BMJ Open Qual.

[159] Navega GBA. Reference nurse in promoting the quality of life of children with hemophilia: parents’ perceptions [Master Thesis]. [Viseu]: Viseu Higher School of Health; 2022. Available from: http://hdl.handle.net/10400.19/7175.

[160] Martins A, Aldiss S, Taylor RM, Gibson F (2022). Care coordination, consistency and continuity: the case of the key worker role in children’s cancer care. Int J Qual Stud Health Well-Being.

[161] Carney JV, Kim H, Duquette K, Guo X, Hazler RJ (2019). Hope as a mediator of bullying involvement and emotional difficulties in children. J Couns Dev.

[162] Charepe Z (2014). Promoting hope in parents of chronically ill children: a mutual aid intervention model.

[163] Henriques NL, Silva JBD, Charepe ZB, Braga PP, Duarte ED (2023). Promoting and threatening factors of Hope in caregivers of children with chronic conditions. Rev Lat Am Enfermagem.

[164] Baile WF, Buckman R, Lenzi R, Glober G, Beale EA, Kudelka AP (2000). SPIKES-A six-step protocol for delivering bad news: application to the patient with cancer. Oncologist.

[165] Aydın A, Savaş EH, Bingöl H, Kebudi R (2024). Taboo words in pediatric oncology: communication experiences of nurses and physicians with dying children and their families. Eur J Oncol Nurs.

[166] Ghoshal A, Muckaden MA, Garg C, Iyengar J, Ganpathy KV, Damani A (2023). Parents’ experiences with prognosis communication in advanced pediatric cancers. Prog Palliat Care.

[167] Kreicbergs U, Nilsson S, Jenholt Nolbris M, Lövgren M (2022). Using communication tools to explore young siblings’ experiences of having a brother or sister with pediatric palliative care needs. Children.

[168] Lagosky S, Bartlett D, Shaw L (2016). Invisible work of using and monitoring knowledge by parents (end-users) of children with chronic conditions. Work.

[169] Malheiro MIDC, Graça M, Figueiredo I (2017). Lay-leds as educators: a self-management educational programme for adolescents with chronic conditions. New Trends Issues Proc Adv Pure Appl Sci.

[170] Van Orne J (2022). Care coordination for children with medical complexity and caregiver empowerment in the process: a literature review. J Spec Pediatr Nurs.

[171] Brooks M, Palau N (2023). Improving the self-efficacy of caregivers of children with seizures using evidence-based practice. J Pediatr Nurs.

[172] Stålberg A, Sandberg A, Larsson T, Coyne I, Söderbäck M (2018). Curious, thoughtful and affirmative-Young children’s meanings of participation in healthcare situations when using an interactive communication tool. J Clin Nurs.

[173] Wong Chung R, Willemen A, Bakker A, Maaskant J, Voorman J, Becher J (2024). The development and validation of the S-scan-parental self-management support (S-scan - PS): a self-reflection tool for child healthcare professionals. Child Care Health Dev.

[174] Miller VA (2018). Involving youth with a chronic illness in decision-making: highlighting the role of providers. Pediatrics.

[175] Quaye AA, Coyne I, Söderbäck M, Hallström IK (2019). Children’s active participation in decision-making processes during hospitalisation: an observational study. J Clin Nurs.

[176] Santos TR. Children/young people with Special Health Needs: promoting effective continuity of care [Master Thesis]. Beja: Polytechnic Institute of Setúbal, Polytechnic Institute of Beja, Polytechnic Institute of Portalegre, Polytechnic Institute of Castelo Branco, University of Évora; 2021. Available from: http://hdl.handle.net/20.500.12207/5496

[177] De Riet LV, Alsem MW, Beijneveld RS, Woensel JBV, Karnebeek CDV. Delineating family needs in the transition from hospital to home for children with medical complexity: part 2, a phenomenological study. In Review; 2023. Available from: https://www.researchsquare.com/article/rs-2526435/v1. 10.1186/s13023-023-02747-w PMC10714565 38082332

[178] Ronan S, Brown M, Marsh L (2020). Parents’ experiences of transition from hospital to home of a child with complex health needs: a systematic literature review. J Clin Nurs.

[179] Neris RR,  Bolis LO, Leite ACAB, de Alvarenga W A, Garcia‐Vivar C, Nascimento LC (2023). Functioning of structurally diverse families living with adolescents and children with chronic disease: A metasynthesis. J Nurs Scholarsh.

[180] Charepe ZB, de Figueiredo MH  JS, da Vieira MM S, fonso Neto  LMV (2011). Discovering hope in families of children with chronic disease through genogram and ecomap. Texto Contexto - Enferm.

[181] Freixo AB dos S. Complex chronic illness in pediatrics: impact on caregiver burden and family functionality [Master Thesis]. Coimbra: Faculty of Medicine, University of Coimbra; 2020. Available from: http://hdl.handle.net/10316/97785

[182] Saimaldaher ZH, Wazqar DY (2020). Relationships between caregiving stress, mental health and physical health in family caregivers of adult patients with cancer: implications for nursing practice. Scand J Caring Sci.

[183] Nogueira AJ, Francisco R (2017). Self-assessment of family quality of life in pediatric palliative care: an exploratory study. Cuid Paliat.

[184] Lutz R, Eibauer C, Frewer A (2022). Prolonged grief as a disease?: Ethics of advance bereavement planning and the case for pediatric palliative care. Ethik Med.

[185] Maia De Sena JG, Melo CDF, Vasconcelos AV, Teixeira LC, Ruiz EM, Alves RSF (2023). The care for oncologic patients undergoing pediatric palliative care and the griefs of a health team. Psicooncología.

[186] Schuelke T, Crawford C, Kentor R, Eppelheimer H, Chipriano C, Springmeyer K (2021). Current grief support in pediatric palliative care. Children.

[187] Widger K, Brennenstuhl S, Nelson KE, Seow H, Rapoport A, Siden H (2023). Intensity of end-of-life care among children with life-threatening conditions: a national population-based observational study. BMC Pediatr.

[188] Spraker-Perlman HL, Aglio T, Kaye EC, Levine D, Barnett B, Berry Carter K (2021). Leveraging grief: involving bereaved parents in pediatric palliative oncology program planning and development. Child Basel Switz.

[189] Vaz EMC, Collet N, Cursino EG, Forte FDS, Magalhães RKBP, Reichert APDS (2018). Care coordination in Health Care for the child/adolescent in chronic condition. Rev Bras Enferm.

[190] Casacio G, Ferrari R, Zilly A, Silva R (2022). Therapeutic itinerary of children with special health care needs: analysis guided by care systems. Rev Gaúcha Enferm.

[191] Bradbury-Jones C, Aveyard H, Herber OR, Isham L, Taylor J, O’Malley L (2022). Scoping reviews: the PAGER framework for improving the quality of reporting. Int J Soc Res Methodol.

[192] United Nations. Transforming our world: the 2030 Agenda for Sustainable Development. UN; 2016. Available from: https://sdgs.un.org/sites/default/files/publications/21252030%20Agenda%20for%20Sustainable%20Development%20web.pdf.

