# Supplementary material for: Health-illness transition processes in children with complex chronic conditions and their parents: a scoping review
Source: BMC Pediatr. 2024 Jul 11;24:446. doi: 10.1186/s12887-024-04919-4 (PMC11238377; doi:10.1186/s12887-024-04919-4)
Supplement: Supplementary file 1 — Supplementary Material 1. [file 12887_2024_4919_MOESM1_ESM.docx]

**Additional File 1**

Final search expression

**Additional File 1**

**Final search expression**

1. Databases with term dictionaries

1. CINAHL Complete

| S1 | TI child* OR TI ( Infant OR Preschool* OR Adolescen* OR "Young Adult" ) OR MH ( Infant OR Child, Preschool OR Child OR Adolescence OR Young Adult ) |
| --- | --- |
| S2 | TI parent* OR TI ( Mothers OR Fathers OR "Parenting Education" OR "Parental Attitudes" OR "Parents of Children with Disabilities" OR "Parent-Child Relations" OR "Parent-Infant Relations" OR "Nuclear Family" OR "Family Relations" ) OR MH ( Parents OR Mothers OR Fathers OR Parenting OR Parenting Education OR Parental Attitudes OR Parents of Children with Disabilities OR Parent-Child Relations OR Parent-Infant Relations OR Nuclear Family OR Family Relations ) |
| S3 | TI caregiv* OR MH Caregivers |
| S4 | S1 OR S2 OR S3 |
| S5 | TI ( "chronic disease" OR "chronic illness" OR "chronic condition" ) OR TI ( "Chronic disease" OR "Noncommunicable Diseases" OR "Communicable diseases" ) OR MH ( Chronic disease OR Noncommunicable Diseases OR Communicable diseases ) |
| S6 | TI ( "Complex chronic condition" OR "Life-limiting condition" OR "Life-threatening condition" ) OR TI ( "Critical illness" ) OR MH Critical illness |
| S7 | TI ( "Long-term conditions" OR "Long-term care" OR "complex care" ) OR TI ( "long term care" ) OR MH Long Term Care |
| S8 | TI "Special health needs" |
| S9 | S5 OR S6 OR S7 OR S8 |
| S10 | TI transition* OR TI ( "Transitional Programs" OR "Transitional Care" OR "Hospital to Home Transition" ) OR MH ( Transitional Programs OR Transitional Care OR Hospital to Home Transition ) |
| S11 | TI continuity OR TI ( "Continuity of Patient Care" OR "After Care" ) OR MH ( Continuity of Patient Care OR After Care ) |
| S12 | TI adapt* OR TI ( "Psychological Adaptation" OR "Physiological Adaptation" OR "Occupational Adaptation" ) OR MH ( Adaptation, Psychological OR Adaptation, Physiological OR Adaptation, Occupational ) |
| S13 | TI diagnos* OR MH Diagnosis |
| S14 | S10 OR S11 OR S12 OR S13 |
| S15 | TI nurs* OR TI ("nursing diagnosis" ) OR MH ( Nurses OR Nursing Diagnosis ) |
| S16 | TI paediatric* OR TI ("paediatric units" OR "paediatric hospitals") OR MH ( Hospitals, Paediatric OR Paediatric Units ) |
| S17 | TI "paediatric nursing" OR TI ( "Paediatric Nursing" OR "Paediatric Nurse Practitioners" OR "Child Care" OR "Infant Care" ) OR MH ( Paediatric Nursing OR Paediatric Nurse Practitioners OR Child Care OR Infant Care ) |
| S18 | TI "paediatric palliative care nursing" OR TI ( "palliative care" OR "hospice nursing" OR "palliative nursing") OR MH ( Palliative Care OR Hospice and Palliative Nursing ) |
| S19 | S15 OR S16 OR S17 OR S18 |
| S20 | S4 AND S9 AND S14 AND S19 |

1. MEDLINE Complete, Cochrane Database of Systematic Reviews® and Cochrane Central Register of Clinical Trials®

| S1 | TI child* OR TI ( Infant OR Preschool* OR Adolescen* OR "Young Adult" ) OR MH ( Infant OR Child, Preschool OR Child OR Adolescent OR Young Adult ) |
| --- | --- |
| S2 | TI parent* OR TI ( Family OR Mothers OR Fathers OR "Parent-Child Relations" ) OR MH ( Parents OR Family OR Mothers OR Fathers OR Parenting OR Parent-Child Relations ) |
| S3 | TI caregiv* OR MH Caregivers |
| S4 | S1 OR S2 OR S3 |
| S5 | TI ( "chronic disease" OR "chronic illness" OR "chronic condition" ) OR TI ( "Noncommunicable diseases" OR "Communicable diseases" )OR MH ( Chronic Disease OR Noncommunicable diseases OR Communicable diseases ) |
| S6 | TI ( "Complex chronic condition" OR "Life-limiting condition" OR "Life-threatening condition" ) |
| S7 | TI ("long-term condition" OR "long-term care" OR "complex care") OR MH Long-Term Care |
| S8 | IT "special health needs" |
| S9 | S5 OR S6 OR S7 OR S8 |
| S10 | TI transition* OR TI ( "Hospital to Home Transition" OR "Transitional Care" OR "Retention in Care" OR "Patient Discharge" ) OR MH ( Hospital to Home Transition OR Transitional Care OR Retention in Care OR Patient Discharge ) |
| S11 | TI continuity OR TI ( "Continuity of Patient Care" OR "After Care" ) OR MH ( Continuity of Patient Care OR After Care ) |
| S12 | TI adapt* OR TI ( "Psychological Adaptation" OR "Physiological Adaptation" OR "Emotional Adjustment" OR "Health Knowledge, Attitudes, Practice" OR "Social Adjustment" ) OR MH ( Adaptation, Psychological OR Adaptation, Physiological OR Emotional Adjustment OR Health Knowledge, Attitudes, Practice OR Social Adjustment ) |
| S13 | TI diagnos* OR MH Diagnosis |
| S14 | S10 OR S11 OR S12 OR S13 |
| S15 | TI nurs* OR TI ("nursing diagnosis" ) OR MH ( Nursing OR Nurses OR Nursing Diagnosis ) |
| S16 | TI paediatric* OR MH ( Paediatrics OR Paediatricians ) |
| S17 | TI "paediatric nursing" OR TI ( "Paediatric nurses" OR "Paediatric Nurse Practitioners" ) OR MH ( Paediatric Nursing OR Nurses, Paediatric OR Paediatric Nurse Practitioners ) |
| S18 | TI "paediatric palliative care nursing" OR TI ( "Palliative Care" OR "Hospice care nursing" OR "Palliative Care Nursing" OR "Respite Care" OR "Episode of Care" ) OR MH ( Palliative Care OR Hospice and Palliative Care Nursing OR Respite Care OR Episode of Care ) |
| S19 | S15 OR S16 OR S17 OR S18 |
| S20 | S4 AND S9 AND S14 AND S19 |

1. Databases without term dictionaries

1. Psychology and Behavioural Sciences Collection

| S1 | TI child* OR AB child* |
| --- | --- |
| S2 | TI parent* OR AB parent* |
| S3 | TI caregiv* OR AB caregiv* |
| S4 | S1 OR S2 OR S3 |
| S5 | TI ( "Chronic disease" OR "Chronic illness" OR "Chronic condition" ) OR AB ( "Chronic disease" OR "Chronic illness" OR "Chronic condition" ) |
| S6 | TI ( "Complex chronic condition" OR "Life-limiting condition" OR "Life-threatening condition" ) OR AB ( "Complex chronic condition" OR "Life-limiting condition" OR "Life-threatening condition" ) |
| S7 | TI ("Long-term conditions" OR "Long-term care" OR "complex care") OR AB ( "Long-term conditions" OR "Long-term care" OR "complex care") |
| S8 | TI "special health needs" OR AB "special health needs" |
| S9 | S5 OR S6 OR S7 OR S8 |
| S10 | TI transition* OR AB transition* |
| S11 | IT continuity OR AB continuity |
| S12 | TI adapt* OR AB adapt* |
| S13 | TI diagnos* OR AB diagnos* |
| S14 | S10 OR S11 OR S12 OR S13 |
| S15 | TI nurs* OR AB nurs* |
| S16 | TI paediatric* OR AB paediatric* |
| S17 | TI "paediatric nursing" OR AB "paediatric nursing" |
| S18 | TI paediatric palliative care nursing OR AB paediatric palliative care nursing |
| S19 | S15 OR S16 OR S17 OR S18 |
| S20 | S4 AND S9 AND S14 AND S19 |

1. Open Aire

| S1 | AB (child) AND AB ( life-limiting condition) |
| --- | --- |

1. RCAAP via b-On

| S1 | TI criança OR AB criança |
| --- | --- |
| S2 | TI ( pais* OR parent* ) OR AB ( pais OR parent* ) |
| S3 | TI cuidad* OR AB cuidad* |
| S4 | S1 OR S2 OR S3 |
| S5 | TI ("doença crónica") OR AB ("doença crónica") |
| S6 | TI ( "Doença Crónica Complexa" OR "Doença Limitadora da vida" OR "Doença Ameaçadora da vida" ) OR AB ( "Doença Crónica Complexa" OR "Doença Limitadora da vida" OR "Doença Ameaçadora da vida" ) |
| S7 | TI ( “doença de longa-duração” OR “cuidados de longa-duração” ) OR AB ( “doença de longa-duração” OR “cuidados de longa-duração” ) |
| S8 | TI “necessidades especiais de saúde” OR AB “necessidades especiais de saúde” |
| S9 | S5 OR S6 OR S7 OR S8 |
| S10 | TI transi* OR AB transi* |
| S11 | TI continu* OR AB continu* |
| S12 | TI adapta* OR AB adapta* |
| S13 | TI diagnost* OR AB diagnost* |
| S14 | S10 OR S11 OR S12 OR S13 |
| S15 | TI enferm* OR AB enferm* |
| S16 | TI pediatr* OR AB pediatr* |
| S17 | TI ( "enfermagem pediátrica" OR “enfermagem de saúde infantil” ) OR AB ( "enfermagem pediátrica" OR “enfermagem de saúde infantil” ) OR KW ( "enfermagem pediátrica" OR “enfermagem de saúde infantil” ) |
| S18 | TI “cuidados paliativos pediátricos” OR AB “cuidados paliativos pediátricos” |
| S19 | S15 OR S16 OR S17 OR S18 |
| S20 | S4 AND S9 AND S14 AND S19 |

Caption

- TI | Search in the "Title" text field
- AB | Search in the "Summary" text field
- MH | Search in the controlled field "MH Exact Subject Heading"
