# Supplementary material for: Health-illness transition processes in children with complex chronic conditions and their parents: a scoping review
Source: BMC Pediatr. 2024 Jul 11;24:446. doi: 10.1186/s12887-024-04919-4 (PMC11238377; doi:10.1186/s12887-024-04919-4)
Supplement: Supplementary file 2 — Supplementary Material 2. [file 12887_2024_4919_MOESM2_ESM.docx]

**Additional File 2**

Final table of search terms

**Additional File 2**

**Final table of search terms**

| **PCC** | **Natural language term** | **MeSH descriptor** | **DeCS descriptor** | **CINAHL descriptor** |
| --- | --- | --- | --- | --- |
| Pop. | Child (child*) | Infant  Child, Preschool  Child  Teenagers  Young Adult | Pre-school  Child  Teenager  Young Adult | Child  Child, Preschool  Infant  Adolescence  Young Adult |
|  | Parents (parent*) | Parents  Family  Mothers  Fathers  Parenting  Parent-Child Relations | Parents  Family  Mums  Dad  Family power  Parent-Child Relationships | Parents  Mothers  Fathers  Parenting  Parenting Education  Parental Attitudes  Parents of Children with Disabilities  Parent-Child Relations  Parent-Infant Relations  Nuclear Family  Family Relations |
|  | Caregiver (caregiv*) | Caregivers | Carers | Caregivers |
|  | Chronic disease  Chronic illness  Chronic condition | Chronic Disease  Non-communicable diseases  Communicable diseases | Chronic illness  Non-communicable diseases  Communicable diseases | Chronic disease  Noncommunicable Diseases  Communicable diseases |
|  | Complex chronic condition  Life-limiting condition  Life-threatening condition | NA  NA  NA | NA  NA  NA | NA  NA  Critical illness |
|  | Long-term conditions  Long-term care  Complex care | Long-Term Care | Long-term care | Long Term Care |
|  | Special health needs | NA | NA | NA |
| Conc. | Transition* | Hospital to Home Transition  Transitional Care  Retention in Care  Patient Discharge | Transition from Hospital to Home  Transitional Care  Retention in Care  Patient discharge | Transitional programmes  Transitional Care  Hospital to Home Transition |
|  | Continuity | Continuity of Patient Care  After Care | Continuity of Patient Care  Convalescent care | Continuity of Patient Care  After Care |
|  | Adaptation (adapt*) | Adaptation, Psychological  Adaptation, Physiological  Emotional Adjustment  Health Knowledge, Attitudes, Practice  Social Adjustment | Psychological Adaptation  Physiological Adaptation  Emotional Adjustment  Health Knowledge, Attitudes and Practices  Social Adjustment | Adaptation, Psychological  Adaptation, Physiological  Adaptation, Occupational |
|  | Diagnosis (diagnos*) | Diagnosis | Diagnosis | Diagnosis |
| Cont. | Nursing (nurs*) | Nursing  Nurses  Nursing Diagnosis | Nursing  Nurses  Nursing Diagnosis | Nurses  Nursing Diagnosis |
|  | Peediatric (paediatric*) | Paediatrics  Paediatricians | Paediatrics  Paediatricians | Hospitals, Paediatric  Paediatric Units |
|  | “Paediatric Nursing” | Paediatric Nursing  Nurses, Paediatric  Paediatric Nurse Practitioners | Paediatric Nursing  Paediatric Nurses  Paediatric Nursing Professionals | Paediatric Nursing  Paediatric Nurse Practitioners  Child Care  Infant Care |
|  | "Paediatric palliative care nursing" / "Palliative care nursing" | Palliative Care  Hospice and Palliative Care Nursing  Respite Care  Episode of Care | Palliative Care  Palliative Care Nursing at the End of Life  Intermittent Care  Periodic care | Palliative Care  Hospice and Palliative Nursing |
