# Supplementary material for: Health-illness transition processes in children with complex chronic conditions and their parents: a scoping review
Source: BMC Pediatr. 2024 Jul 11;24:446. doi: 10.1186/s12887-024-04919-4 (PMC11238377; doi:10.1186/s12887-024-04919-4)
Supplement: Supplementary file 3 — Supplementary Material 3. [file 12887_2024_4919_MOESM3_ESM.docx]

**Additional File 3**

Exclusion of articles after full text analysis – reasons and references

**Additional File 3**

**Exclusion of articles after analysing the full text – reasons and references**

| **Population \| Children with non-complex or acute illnesses** | **31** |
| --- | --- |
| 1. Ajbar A, Cross E, Matoi S, Hay CA, Baines LM, Saunders B, et al. Diagnostic Delay in Pediatric Inflammatory Bowel Disease: A Systematic Review. Dig Dis Sci. 2022;67(12):5444–54. 2. Anand V, Zeft AS, Spalding SJ. Screening for Behavioral Risks: A Precision Healthcare Driven Approach for Chronic Pain Evaluation in Pediatric Specialty Care. Stud Health Technol Inform. 2017;245:275–9. 3. Anderzén-Carlsson A. CHARGE Syndrome—A Five Case Study of the Syndrome Characteristics and Health Care Consumption During the First Year in Life. J Pediatr Nurs. 1 de fevereiro de 2015;30(1):6–16. 4. Antão JM, Portugal MGC de C, Silva CF da. O ajustamento de crianças com doença crônica. 2019 [citado 1 de janeiro de 1AD]; 5. Vockell ALB, Wimberg J, Britto M, Nye A. Using a Parent Coordinator to Support the Role of the Pediatric Nurse Practitioner in Care Coordination. J Pediatr Health Care Off Publ Natl Assoc Pediatr Nurse Assoc Pract. 2018;32(1):36–42. 6. Chaves AVC. Adaptação da criança à doença crónica gastrointestinal, com necessidades especiais de alimentação. 2014 [citado 1 de janeiro de 6AD]; 7. da Silva Costa J, Caldeira dos Santos MLS. GRUPO DE ADOLESCENTES HOSPITALIZADOS COM DOENÇA CRÔNICA NÃO TRANSMISSÍVEL COMO TECNOLOGIA DE CUIDADO EM ENFERMAGEM. J Nurs UFPE Rev Enferm UFPE. fevereiro de 2016;10(2):508–14. 8. Desai PP, Rivera AT, Backes EM. Latino Caregiver Coping With Children’s Chronic Health Conditions: An Integrative Literature Review. J Pediatr Health Care Off Publ Natl Assoc Pediatr Nurse Assoc Pract. 2016;30(2):108–20. 9. Drutchas A, Anandarajah G. Spirituality and coping with chronic disease in pediatrics. R I Med J. 2014;97(3):26–30. 10. Dushnicky MJ, Beattie KA, Cellucci T, Heale L, Zachos M, Sherlock M, et al. Pediatric Patients With a Dual Diagnosis of Inflammatory Bowel Disease and Chronic Recurrent Multifocal Osteomyelitis: A Single-Centre Case Series - Response to Letter to the Editor. J Pediatr Gastroenterol Nutr. maio de 2022;74(5):e129–e129. 11. Emerson ND, Morrell HER, Neece C, Tapanes D, Distelberg B. Longitudinal Model Predicting Self‐Concept in Pediatric Chronic Illness. Fam Process. 2019;58(1):100–13. 12. Freitas CIL. Crenças sobre a doença em famílias com doença crónica pediátrica: Estudo preliminar da versão portuguesa do instrumento Iceland-Family Illness Beliefs Questionnaire. Illn Beliefs Fam Pediatr Chronic Dis Prelim Study Port Version Icel-Fam Illn Beliefs Quest Instrum [Internet]. 2021 [citado 1 de janeiro de 12AD]; 13. Gehring JMK. Family Management, Risk and Protective Factors, and Quality of Life with Pediatric Chronic Conditions. Fam Manag Risk Prot Factors Qual Life Pediatr Chronic Cond. janeiro de 2017;(Ph.D.):1–1. 14. Hoying C, Lecher WT, Mosko DD, Roberto N, Mason C, Wade Murphy S, et al. «On the Scene». Nurs Adm Q. janeiro de 2014;38(1):27–54. 15. Hytiris M, Johnston D, Mullen S, Smyth A, Dougan E, Rodie M, et al. Experience of health care at a reference centre as reported by patients and parents of children with rare conditions. Orphanet J Rare Dis. 2021;16(1):65. 16. Kluthe C, Isaac DM, Hiller K, Carroll M, Wine E, van Manen M, et al. Qualitative Analysis of Pediatric Patient and Caregiver Perspectives After Recent Diagnosis With Inflammatory Bowel Disease. J Pediatr Nurs. 2018;38:106–13. 17. Massimo L, Rossoni N, Mattei F, Bonassi S, Caprino D. Needs and expectations of adolescent in-patients: the experience of Gaslini Children’s Hospital. Int J Adolesc Med Health. fevereiro de 2016;28(1):11–7. 18. Mendes T, Crespo C, Austin J. Family Cohesion and Adaptation in Pediatric Chronic Conditions: The Missing Link of the Family’s Condition Management. J Child Fam Stud. setembro de 2016;25(9):2820–31. 19. Moreira H, Carona C, Silva N, Frontini R, Bullinger M, Canavarro MC. Psychological and quality of life outcomes in pediatric populations: a parent-child perspective. J Pediatr. novembro de 2013;163(5):1471–8. 20. Moura LMCH. Qualidade de Vida na Doença Inflamatória Intestinal em Idade Pediátrica. 2019 [citado 1 de janeiro de 5AD]; 21. Peixoto RFS. Impacto da doença crónica pediátrica na qualidade de vida dos pais e satisfação com os cuidados de saúde. Impact Pediatr Chronic Dis Parent’s Qual Life Healthc Satisf [Internet]. 2021 [citado 1 de janeiro de 12AD]; 22. Perry Caldwell E, Killingsworth E. The health literacy disparity in adolescents with sickle cell disease. J Spec Pediatr Nurs. 2021;26(4):1–6. 23. Ramos de Miranda F, Ivo ML, Ferraz Teston E, Trindade Lino IG, Aparecida Mandetta M, Marcheti MA. Families’ experience in managing children with sickle cell anemia: implications for care. Rev Enferm UERJ. janeiro de 2020;28:1–6. 24. Rohan JM, Verma T. Psychological Considerations in Pediatric Chronic Illness: Case Examples. Int J Environ Res Public Health [Internet]. 2020 [citado 1 de janeiro de 3AD];17(5). 25. Sachdeva A, Gunasekaran V, Ramya HN, Dass J, Kotwal J, Seth T, et al. Consensus Statement of the Indian Academy of Pediatrics in Diagnosis and Management of Hemophilia. Indian Pediatr. 2018;55(7):582–90. 26. Svavarsdottir EK, Sigurdardottir AO, Tryggvadottir GB. Strengths-oriented therapeutic conversations for families of children with chronic illnesses: findings from the Landspitali university hospital family nursing implementation project. J Fam Nurs. 2014;20(1):13–50. 27. Szulczewski L, Mullins LL, Bidwell SL, Eddington AR, Pai ALH. Meta-Analysis: Caregiver and Youth Uncertainty in Pediatric Chronic Illness. J Pediatr Psychol. 2017;42(4):395–421. 28. Teixeira VMM. Integração da doença crónica pediátrica na vida familiar: estudo preliminar da versão portuguesa do instrumento «Family Adaptation to Chronic Illness Questionnaire». Integrating Pediatr Chronic Illn Fam Life Prelim Study Port Version Instrum Fam Adapt Chronic Illn Quest [Internet]. 2021 [citado 1 de janeiro de 12AD]; 29. Werner H, Balmer C, Lehmann P. Posttraumatic stress and health-related quality of life in parents of children with cardiac rhythm devices. Qual Life Res. 2019;28(9):2471–80. 30. National Association of Pediatric Nurse Practitioners. NAPNAP Position Statement. Position Statement on Pediatric Health Care/Medical Home: Key Issues on Care Coordination, Transitions, and Leadership. J Pediatr Health Care. 2016;30(2):A17–9. 31. Phillips CD, Truong C, Kum HC, Nwaiwu O, Ohsfeldt R. Post-acute care for children with special health care needs. Disabil Health J. 2018;11(1):49–57. | |
| **Concept \| Exclusive identification of transition processes other than health-disease** | **21** |
| 1. Amirnovin R, Aghamohammadi S, Riley C, Woo MS, Del Castillo S. Analysis of a Pediatric Home Mechanical Ventilator Population. Respir Care. maio de 2018;63(5):558–64. 2. Ardenghi C, Vestri E, Costanzo S, Lanfranchi G, Vertemati M, Destro F, et al. Congenital Esophageal Atresia Long-Term Follow-Up—The Pediatric Surgeon’s Duty to Focus on Quality of Life. Children. março de 2022;9(3):331. 3. Daly A, Lewis RW, Vangile K, Masker KW, Effinger KE, Meacham LR, et al. Survivor clinic attendance among pediatric- and adolescent-aged survivors of childhood cancer. J Cancer Surviv. fevereiro de 2019;13(1):56–65. 4. Lopes FG. Sobre dignidade e morte: a experiência de profissionais em cuidados paliativos na realidade de um serviço de oncologia pediátrica do Brasil. 2015 [citado 1 de janeiro de 1AD]; 5. Dellon EP, Helms SW, Hailey CE, Shay R, Carney SD, Schmidt HJ, et al. Exploring knowledge and perceptions of palliative care to inform integration of palliative care education into cystic fibrosis care. Pediatr Pulmonol. 2018;53(9):1218–24. 6. dos Santos RA, Moreira MCN. Resilience and death: the nursing professional in the care of children and adolescents with life-limiting illnesses. Cienc Saude Coletiva. 2014;19(12):4869–78. 7. English L, Kumbakumba E, Larson CP, Kabakyenga J, Singer J, Kissoon N, et al. Pediatric out-of-hospital deaths following hospital discharge: a mixed-methods study. Afr Health Sci. 2016;16(4):883–91. 8. Franco TS dos R. A morte da criança/do jovem. Interv Enferm Espec No Luto Parent [Internet]. 2014 [citado 1 de janeiro de 1AD]; 9. Hinson AP, Rosoff PM. Where Children Die. [Internet]. Vol. 55. 2016. p. 106. 10. Jaaniste T, Coombs S, Donnelly T, Kelk N, Beston D. Risk and Resilience Factors Related to Parental Bereavement Following the Death of a Child with a Life-Limiting Condition. Children. 2017;4(11):96. 11. Jennings V, Nicholl H. Bereavement support used by mothers in Ireland following the death of their child from a life-limiting condition. Int J Palliat Nurs. abril de 2014;20(4):173–8. 12. Lemke M, Kappel R, McCarter R, D’Angelo L, Tuchman LK. Perceptions of Health Care Transition Care Coordination in Patients With Chronic Illness. Pediatrics. 2018;141(5):1–8. 13. Linton JM, Reichard E, Peters A, Albertini LW, Miller-Fitzwater A, Poehling K. Enhancing Resident Education and Optimizing Care for Children With Special Health Care Needs in Resident Continuity Clinics. Acad Pediatr. 2018;18(4):366–9. 14. Oeffinger KC, Stratton KL, Hudson MM, Leisenring WM, Henderson TO, Howell RM, et al. Impact of Risk-Adapted Therapy for Pediatric Hodgkin Lymphoma on Risk of Long-Term Morbidity: A Report From the Childhood Cancer Survivor Study. J Clin Oncol Off J Am Soc Clin Oncol. 10 de julho de 2021;39(20):2266–75. 15. Oliveira L, Coelho J, Ferreira R, Nunes T, Saianda A, Pereira L, et al. Long-Term Home Oxygen Therapy in Children: Evidences and Open Issues. Oxigenoter Domic Longa Duraç Na Criança Evidências E Questões Em Aberto [Internet]. 2014; 16. Pimenta RJV. Cuidados paliativos em oncologia pediátrica: Necessidades, preocupações e dificuldades dos pais e crianças/adolescentes na ótica dos profissionais [Internet] [Dissertação de Mestrado]. [Braga]: Instituto de Educação da Universidade do Minho; 2013. Disponível em: https://hdl.handle.net/1822/28903 17. Smith P, Teasdale E, Sheppard-Law S. Parents’ experience of extended viewing in a paediatric hospice: a qualitative study. Int J Palliat Nurs. 2023;29(1):34–42. 18. Williams NA, Brik AB, Petkus JM, Clark H. Pediatric psychosocial care: Historical context and a theoretically informed practice model. Child Youth Serv Rev. dezembro de 2019;107:N.PAG-N.PAG. 19. Zimmermann K. Paediatric End-of-LIfe CAre Needs in Switzerland (PELICAN) : Current end-of-life care practices and the persepctives of bereaved parents. 2016; 20. Madureira AF, Moreira MCN, Sá MC de. Physiotherapy for New Actors: Disputes and Innovations in Care for Chronically Ill Children. Cienc Saude Coletiva. 2019;24(5):1743–52. 21. Noyes J, Edwards RT, Hastings RP, Hain R, Totsika V, Bennett V, et al. Evidence-based planning and costing palliative care services for children: novel multi-method epidemiological and economic exemplar. BMC Palliat Care. 2013;12(1):18. | |
| **Type of Evidence \| Inadequate type of evidence** | **23** |
| 1. Coimbra CSM. Preparação do regresso a casa: Cuidar a criança em diálise peritoneal e familia [Internet]. Lisboa: Escola Superior de Enfermagem de Lisboa; 2014 [citado 1 de janeiro de 1AD]. 2. Costa AS de J. Relatório de estágio. 2022 [citado 1 de janeiro de 7AD]; 3. Freitas AFM de. O brincar na promoção do desenvolvimento da criançacom necessidades especiais de saúde. Contrib Enferm Espec [Internet]. 2020 [citado 1 de janeiro de 1AD]; 4. Jesus MI de S e C. Cuidados Especializados à Criança em Cuidados Paliativos: Importância da Equipa de Suporte Integrado Pediátrico [Internet]. Setúbal: Escola Superior de Saúde do Instituto Politécnico de Setúbal; 2019 [citado 18 de novembro de 2022]. 5. Marcelino M. Promover a esperança nos pais de crianças e jovens em cuidados paliativos pediátricos [Internet]. Lisboa: Universidade Católica Portuguesa; 2017. 6. Mendonça TRI. A invisibilidade do cuidado emocional à criança com doença crónica. 2016 [citado 1 de janeiro de 1AD]; 7. Mota LM da CRN. Cuidar na transição. Um Programa Interv Enferm Aos Pais Criança Com Atrofia Muscular Espinhal Tipo I [Internet]. 2014 [citado 1 de janeiro de 1AD]; 8. Nganda ÂC. Relatório de estágio em reabilitação neurológica: intervenção da fisioterapia em crianças com necessidades especiais. 2014 [citado 1 de janeiro de 11AD]; 9. Ribeiro CP de SPTG. Preparação e acompanhamento da criança e jovem a vivenciar as transições associadas a um transplante renal. Um Programa Interv Enferm [Internet]. 2017 [citado 1 de janeiro de 1AD]; 10. Santos PE dos. O cuidado de enfermagem promotor da adaptação do adolescente à doença crónica. 2020 [citado 1 de janeiro de 1AD]; 11. Santos RR. Continuidade de cuidados em pediatria: Ser criança com doença crónica no domicílio: Uma resposta pediátrica. 2019 [citado 1 de janeiro de 5AD]; 12. Santos TR. A criança/jovem com Necessidades de Saúde Especiais: promoção de uma efetiva continuidade de cuidados. 2021; 13. Torgal JRMG. Cuidar da criança e família com doença crónica em fim de vida. Estratégias Gest Emocional [Internet]. 2017 [citado 1 de janeiro de 1AD]; 14. Flora MC. O enfermeiro e a criança com diabetes Tipo 1 na escola. 2019 [citado 1 de janeiro de 11AD]; 15. Frazão I, Reis A, Candido A, Figueiredo M do C (preferencial), Pascoal D, Jorge M, et al. Processo de transição saúde-doença na adolescência: apreciação de uma família. 2022 [citado 1 de janeiro de 1AD]; 16. Lomba M de LL de F. Padrões de qualidade dos cuidados de enfermagem pediátrica. Gestão da doença crónica. 2013 [citado 1 de janeiro de 5AD]; 17. Pires M do CCM, Menino EP da SG, Silva AMM, Arrue AM, Figueiredo M do CAB de. Rastreio de crianças com necessidades de saúde especiais em contexto escolar: estratégias em contexto de pandemia por COVID-19. 2022 [citado 1 de janeiro de 9AD]; 18. Silva MG, Geraldo SSA, Pires A, Fernandes V, Pimentel MH. Estimativa das necessidades de equipas intra-hospitalares de suporte em cuidados paliativos pediátricos em Portugal. 2022 [citado 1 de janeiro de 1AD]; 19. Fahlberg B. Integrating supportive and palliative care for young adults with serious illnesses. Nursing (Lond). junho de 2016;46(6):12–4. 20. Kellerman SE, Sugandhi N, Luo C, McClure C, Yogev R. Addressing and improving the continuum of care for HIV-affected children: challenges and solutions. AIDS. 2013;27:S135–7. 21. Melo ASI de E Universidade do Minho, Caires SI de E Universidade do Minho, Machado MI de E Universidade do Minho, Pimenta RI de E Universidade do Minho. Pediatria oncológica: o olhar dos profissionais hospitalares em torno das vivências das crianças, adolescentes e seus pais. 2013 [citado 1 de janeiro de 10AD]; 22. Kichler JC, Kaugars AS. Topical Review: Applying Positive Development Principles to Group Interventions for the Promotion of Family Resilience in Pediatric Psychology. J Pediatr Psychol. 2015;40(9):978–80. 23. Pickles DM, Lihn SL, Boat TF, Lannon C. A Roadmap to Emotional Health for Children and Families With Chronic Pediatric Conditions. Pediatrics [Internet]. 2020 [citado 1 de janeiro de 2AD];145(2). | |
| **Population \| Population over 21 (excluding parents)** | **7** |
| 1. Graça NM. Fisioterapia em crianças com necessidades especiais: relatório de estágio em reabilitação neurológica. 2015 [citado 1 de janeiro de 10AD]; 2. Graham RJ, Amar-Dolan LR, Roussin CJ, Weinstock PH. Bridging the Stressful Gap Between ICU and Home: Medical Simulation for Pediatric Patients and Their Families. Pediatr Crit Care Med. abril de 2019;20(4):e221–4. 3. Freixo AB dos S. Doença crónica complexa em pediatria: impacto na sobrecarga dos cuidadores e na funcionalidade familiar. Pediatr Complex Chronic Cond Impact Caregiv Burd Fam Funct [Internet]. 2020 [citado 1 de janeiro de 6AD]; 4. Johnston EE, Currie ER, Chen Y, Kent EE, Ornstein KA, Bhatia S, et al. Palliative Care Knowledge and Characteristics in Caregivers of Chronically Ill Children. J Hosp Palliat Nurs JHPN Off J Hosp Palliat Nurses Assoc. 2020;22(6):456–64. 5. Guerra D. Cuidar do adolescente com doença hemato-oncológica hospitalizado. Interv Enferm No Processo Transição [Internet]. 2018 [citado 1 de janeiro de 1AD]; 6. Paixão SMB. Cuidados Paliativos Pediátricos: Necessidades formativas, coping e perceção de esperança dos profissionais de saúde [Internet]. Viseu: Escola Superior de Saúde do Instituto Politécnico de Viseu; 2018 [citado 18 de novembro de 2022]. 7. Clark JK, Fasciano K. Young Adult Palliative Care: Challenges and Opportunities. Am J Hosp Palliat Med. fevereiro de 2015;32(1):101–11. | |
| **Concept \| Process of transition from healthcare in a pediatric environment to an adult environment** | **4** |
| 1. Celona CA. Measuring Acuity and Patient Progress for Youth With Special Health Care Needs in Transition Care Utilizing Nursing Outcomes. J Pediatr Nurs. 2015;30(5):e15–8. 2. Esposito S, Rosafio C, Antodaro F, Argentiero A, Bassi M, Becherucci P, et al. Use of Telemedicine Healthcare Systems in Children and Adolescents with Chronic Disease or in Transition Stages of Life: Consensus Document of the Italian Society of Telemedicine (SIT), of the Italian Society of Preventive and Social Pediatrics (SIPPS), of the Italian Society of Pediatric Primary Care (SICuPP), of the Italian Federation of Pediatric Doctors (FIMP) and of the Syndicate of Family Pediatrician Doctors (SIMPeF). J Pers Med [Internet]. 2023 [citado 1 de janeiro de 1AD];13(2). 3. Schraeder K, Allemang B, Felske AN, Scott CM, McBrien KA, Dimitropoulos G, et al. Community based Primary Care for Adolescents and Young Adults Transitioning From Pediatric Specialty Care: Results from a Scoping Review. J Prim Care Community Health. 24 de março de 2022;13:1–29. 4. van Staa A, Sattoe JNT, Strating MMH. Experiences with and Outcomes of Two Interventions to Maximize Engagement of Chronically Ill Adolescents During Hospital Consultations: A Mixed Methods Study. J Pediatr Nurs. 9 de outubro de 2015;30(5):757–75. | |
| **Type of Evidence \| Foreign Language** | **3** |
| 1. Kremeike K, Mohr A, Kampschulte R, Bergmann J, Beil S, Neuhaus U, et al. [Network for Oncological Advisory Service (NOF) - a Pilot Project for (Long-Term) Follow-Up Care of Pediatric Cancer Patients]. Klin Padiatr. 2016;228(6):325–31. 2. Seliner B, Wattinger A, Spirig R. [Experiences and needs of parents of hospitalised children with disabilities and the health professionals responsible for the child’s health-care – A systematic review]. Pflege. 2015;28(5):263–76. 3. Tellier H, Colson S, Gentile S. [Improving the management of children with type 1 diabetes and their families: What role for the advanced practice nurse, coordinator of complex care pathways? A qualitative, exploratory study using semi-directed interviews]. Rech Soins Infirm. 2019;(136):80–9. | |
| **Type of Evidence \| Repetition of a study already included in another evidence type** | **3** |
| 1. Alves JMN de O. Oportunidades de parceria no cuidar de crianças com necessidades especiais de saúde : a perspetiva dos pais. 2015 [citado 1 de janeiro de 5AD]; 2. Dunbar H. ‘Place Bonding’: Parents’ Journeys Towards a Sense of Rootedness in Children’s Hospice Care. 2016 [citado 1 de janeiro de 11AD]; 3. Paixão SMB. Cuidados Paliativos Pediátricos: Necessidades formativas, coping e perceção de esperança dos profissionais de saúde [Internet]. Viseu: Escola Superior de Saúde do Instituto Politécnico de Viseu; 2018 [citado 18 de novembro de 2022]. | |
| **Total** | **92** |
