# Supplementary material for: Health-illness transition processes in children with complex chronic conditions and their parents: a scoping review
Source: BMC Pediatr. 2024 Jul 11;24:446. doi: 10.1186/s12887-024-04919-4 (PMC11238377; doi:10.1186/s12887-024-04919-4)
Supplement: Supplementary file 4 — Supplementary Material 4. [file 12887_2024_4919_MOESM4_ESM.docx]

**Additional File 4**

Summary of the characteristics and results of the studies included in the review

| **Authors** | **Year and Country** | **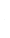Type of study** | **Objective** | **Methodology** | **Results** |
| --- | --- | --- | --- | --- | --- |
| Aguiar et al. [92] | 2021  Brazil | Primary - Qualitative | To identify the main challenges faced by children with type 1 diabetes mellitus (DM1) and describe their coping strategies | Semi-structured interview with children with DM1 between the ages of 8 and 11 receiving outpatient care at a university hospital | - The main challenges are the need to manage the therapeutic regime, the beliefs associated with chronic illness, the changes in daily life and the need for frequent clinical follow-up.  - Depression, non-acceptance of the disease, social isolation, fear and anxiety are negative responses when coping is not implemented.  - Strategies that facilitate coping are family therapy, family involvement in care and therapeutic education using recreational resources.  - The presence of knowledge and skills about the therapeutic regime, as well as the ability to recognise signs of severity, is a positive response pattern when coping is implemented. |
| Alves & Fontaine [58] | 2016  Portugal | Primary - Qualitative | Exploring the emotional needs and difficulties of parents whose child is suffering from an illness that requires pediatric palliative care (PPC) | Semi-structured interviews with 21 parents, the content of which was subjected to content analysis according to Bardin's approach. | - The diagnosis of a disease that requires PPC in the child generates feelings, emotions and difficulties associated with the disease process, and it is necessary to develop strategies to help parents adjust to it.  - The presence of negative emotions such as fear, uncertainty, and the confrontation with the approaching finitude, combined with the special health needs (SHN) (uncontrolled symptoms, life-support devices, recurrent hospitalisations, etc.), inadequate communication and the perception that the parents' opinion is devalued, make the parents' transition process difficult. Lack of family support and low levels of government support for these children and families are also identified as factors that contribute negatively to this process.  - Effective family support, together with a positive relationship between the couple, the support provided by siblings, leisure activities not focused on the illness process, maintaining work activities and the family's acceptable economic capacity are all factors that facilitate the transitional process. Availability of the health care team and social support are also mentioned as positive strategies in this order. |
| Alves, Amendoeira & Charepe [57] | 2017  Portugal | Primary - Qualitative | Understanding the experience of care partnership in parents of children with special health needs (SHN) | Unstructured interviews with 10 parents of children with SHN and CCC, resulting in narratives that were subjected to content analysis. | - The experience of partnership between parents of children with SHN and professionals was found in two dynamics - teaching and training parents in relation to the SHN demonstrated by the child (including learning, communication and transmission of information, the existence of a reference nurse, involvement in care and the relationship with nurses) and decision-making (regarding learning, providing care and supporting the parents' decision).  - Through partnership, parents of children with SHN optimise their parental role and increase their empowerment. |
| Andrade et al. [105] | 2020  Portugal | Case Study / Project | To review the rationale inherent in a palliative approach at home, through the presentation of a clinical case | Data collection with unspecified methodology on a 14-year-old child with palliative needs | - The home-based PPC approach, considering a multidisciplinary intervention plan with coordination between in-hospital and out-of-hospital teams, is seen as a solution to promote well-being, as well as symptomatic control of the child and a reduction in hospitalisations since its implementation.  - The dynamics associated with the caregivers learning about the therapeutic regimen facilitate the comfort of the child with palliative needs, along with family support.  - The child's lack of symptom control, economic difficulties and non-recognition of the informal caregiver are factors that make it difficult to control the child's palliative needs. |
| Antolick et al. [59] | 2020  USA | Case Study / Project | Improving the communication of post-discharge care objectives for CMC in a paediatric specialty setting based on a quality improvement project | Drawing up a flowchart and support tool for decision-making, using procedural optimisation strategies, applied to families of CMC hospitalised for more than three months. | - Systematising the process of communicating post-discharge goals to families of CMC is an individualised intervention with the potential to support the transition between hospital and community settings.  - The use of technology was seen as facilitating the development of processes for communicating post-discharge care goals in this context, rather than the high costs, resistance from professionals and parental difficulties in understanding the long-term needs of these children, which were seen as barriers. |
| Baird et al. [87] | 2016  USA | Primary - Qualitative | Exploring the continuity of nursing care for children with complex chronic illness (CCC) in a paediatric intensive care unit (PICU) from the perspective of parents and nurses | Interviews and observation of seven parents and twelve nurses associated with a PICU, as well as documentary analysis of institutional material. The data was subjected to situational analysis. | - Promoting the individualisation of care through the reference nurse’ methodology makes it possible to increase nurses' knowledge of each child's particularities, facilitating the provision of care.  - Continuity lists generate positive responses from parents, such as relief, confidence and a perception of quality in the care provided, but at the same time these lists are identified as challenging for professionals, particularly in terms of the loss of learning opportunities and difficulties in the associated emotional management.  - From the parents' point of view, the absence of a reference nurse and the consequent assignment of different nurses to each shift can make it difficult for professionals to get to know the child's needs and can increase insecurity, uncertainty and mistrust towards them. |
| Barata [60] | 2016  Portugal | Primary - Quantitative | To determine the level of quality of life of children/young people with diabetes; to identify the sociodemographic variables that interfere with the quality of life of children/young people with diabetes and to relate the child's/young person's satisfaction with the diabetes consultation to their quality of life. | A questionnaire focussed on sociodemographic characteristics, satisfaction and quality of life, applied to 135 children and young people between the ages of 8 and 18 followed up in a diabetes clinic. | - The relational and communicational dynamics of the nurses in the diabetes consultation is a determining factor in the satisfaction of children and young people with this service.  - In turn, the satisfaction of children and young people with diabetes with the advice they receive plays an important role in promoting quality of life. |
| Bennett [61] | 2020  United Kingdom | Primary - Qualitative | Deepen understanding of the contextual and relational complexity of advanced care planning for parents of children with life-limiting (LLI) or life-threatening illnesses (LTI) | Documentary analysis and semi-structured interviews with 13 parents of children with LLI/LTI, the content of which was analysed using a permanent comparative approach. | - Reconstructing the meaning of the advanced care plan with parents is seen as a facilitator, by providing tools that enable a differentiated understanding of the issue and communication based on reconciling multiple tensions, building trust and giving control to parents of children with LLI/LTI.  - By reconstructing the meaning of the advanced care plan, parents of children with LLI/LTI optimise their ability to adapt, adjusting their thoughts, beliefs and expectations to the child's clinical situation. This is also demonstrated by greater openness in the family system, which in turn enables greater participation in care. |
| Berry et al. [118] | 2014  USA | Narrative Literature Review | To review the evidence related to the hospital discharge process and to present an action model for discharging hospitalised children. | Narrative review of scientific literature and legislative instruments related to the subject, which have informed the production of a model of care for the child's discharge from admission. | - The lack of standards of care at hospital discharge has the potential to jeopardise the quality of this transition, and an intervention that promotes the involvement of the child and family in this process is essential.  - A care model based on the specification of discharge objectives, which can be modified throughout the hospital stay, as well as the assessment of healthcare needs for continuity of care after discharge, facilitate the management of this transitional process.  - The implementation of this model is potentially associated with healthy response patterns, associated with the optimisation of the child's and parents' knowledge and skills in managing the therapeutic regime, the quality of care provided in the home environment and the recognition of alarm signals. |
| Branowicki et al. [107] | 2016  USA | Primary - Qualitative | To describe the daily issues and challenges faced by carers during the transition from hospital to home following their child's cancer diagnosis. | A retrospective study analysing the content of nursing records of home visits to children recently diagnosed with cancer. | - During the transition to home for a child with a recent diagnosis of cancer, different patterns of response co-exist. The most positive are associated with mastery in developing the caregiver role and maintaining well-being. However, there are also negative patterns associated with inadequate training and mastery, diminished well-being, and a recurrent need for clinical follow-up.  - Issues related to the complexity of symptom control and treatment regimens, as well as insurance problems, housing difficulties and interruptions in clinical follow-up are seen as barriers to the transition from hospital to home.  - The involvement of a reference nurse, who assesses the child, collaborates in planning and adapting care to the home environment, provides therapeutic education and manages operational constraints, is the main factor facilitating the transition. |
| Brunetta et al. [139] | 2022  The Netherlands | Systematic Literature Review | Assessing the appropriate age for implementing advanced care planning for children with LLI | Literature search in CINAHL, Embase and MEDLINE. A total of 31 articles were included in the review, the results of which were subjected to narrative analysis. | - Eighteen advanced care planning tools were found, most of which were aimed at adolescents and young adults.  - Most of the tools focus primarily on the communication and relationship aspects with children and parents (including topics such as hope, wishes, goals and preferences for care and treatment, and the needs of the family).  - The appropriate age to start advance care planning can be influenced by the willingness and ability to participate, the development of a social identity in adolescence and legal responsibilities. |
| Cady et al. [94] | 2015  USA | Primary - Quantitative | Present preliminary evidence to introduce a nurse-led telehealth-based care coordination model in a residential CMC facility | Randomised controlled clinical trial with a control group (n=55) and two intervention groups (n=54/54) (one with a telephone intervention and the other with the addition of a video intervention). | - A nurse-led, telehealth-based care coordination model has been identified as an advantageous solution for CMC.  - The existence of a nurse who is familiar with the clinical history and particularities of each child, who has an advanced understanding of the management of the chronic condition and who promotes a partnership with the parents, was identified as a strategy that facilitates the coordination of care.  - The implementation of this model leads to better coordination of care for CMC between professionals, a reduction in stress for carers, a better balance between care dynamics and professional life, as well as a reduction in the number of follow-up appointments required. |
| Caicedo [51] | 2016  USA | Primary - Quantitative | Describe child health, functional outcomes and utilisation of health services associated with technology-dependent CMC | A longitudinal descriptive study, with a questionnaire applied to a convenience sample of 84 parents of technology-dependent CMC. | - Technology-dependent CMCs have various needs, from those related to self-care and functionality (eating, mobility, hygiene, clothing, toilet use) to emotional issues (prevalence of negative feelings such as sadness, fear and worry) and social problems (difficulty socialising and interpersonal relationships).  - Their therapeutic regime often involves polymedication, the use of technological devices to support vital functions and the need for health monitoring by multiple professionals (doctors, nurses and therapists).  - The use of technological medical devices and the existence of a nurse responsible for coordinating care are identified as factors that facilitate therapeutic management in the CMC.  - Positive functional outcome patterns reported for these technology-dependent CMC are improved independence and functionality, as well as health-seeking behaviour and adherence to therapy, while functional limitation, diminished physical condition and the child's obnoxious state are described as negative functional outcome patterns. |
| Carter et al. [119] | 2016  United Kingdom | Primary - Qualitative | Exploring nurses' intervention in the transition between hospital and home for SHN | Appreciative enquiry through interviews with 9 nurses and 37 local stakeholders, the results of which were subjected to thematic, interpretative and collaborative analysis. | - Appreciation and knowledge of the child's and family's home space is crucial in the process of transitioning children with SHN from hospital to home.  - The implementation of an intervention and support programme for the transition between the hospital and the child and family's home has reduced the fragmentation of care and the challenges faced in this context. |
| Carvalho [62] | 2014  Portugal | Case Study / Project | To assess and intervene in the families of children and adolescents with chronic illnesses (CI) enrolled in a Family Health Unit. | Community intervention project with the implementation of a family care consultation and a mutual support group for families. | - Interaction, sharing experiences and difficulties, collaborative problem-solving and hope are all factors that facilitate assessment and intervention with children with CI.  - The implementation of a nursing consultation and a family support group are described as important solutions for the families of children with CI, with significant benefits for the parental role, namely by reducing the saturation and conflict associated with the role. |
| Carvalho et al. [139] | 2019  Brazil | Primary - Quantitative | Evaluating the effectiveness of home care services in the paediatric field. | A retrospective descriptive study based on an analysis of the clinical files of children with palliative needs admitted to home care (group 1) and hospitalised with an indication for this service (group 2). | - The presentation of a home-based long-term care solution is seen as effective for the continuity of the therapeutic approach to children with palliative needs and their families.  - With this solution, there are positive response patterns, such as a reduction in the number of procedures required and the rate of infections, as well as a reduction in the number of hospitalisations. |
| Ciobanu & Preston [63] | 2021  Romania | Primary - Qualitative | Advancing our understanding about the experiences of children with LLI/LTI | Interviews were carried out with 10 dyads made up of children aged between 14 and 17 with LLI/LTI and their mothers, and the results were subjected to thematic analysis. | - When faced with LLI/LTI, negative response patterns persist in the presence of mismatches between children and their families - low awareness of death, depression, anxiety and sadness in children; avoidance behaviour and conspiracy of silence in parents.  - For the children, unclear and closed family communication on issues related to the illness makes it difficult to live with it, while open communication and support from the family, socialisation, distraction, spirituality and optimism were identified as factors that make it easier.  - In the case of parents, they consider that the perception of peer pressure felt by children to show that they can be the same is the main barrier to living with the illness, to which spirituality makes a positive contribution. Symptomatic uncontrol was perceived as a barrier by both children and parents.  - When barriers to healthy living are overcome, coping and independence were the positive responses emphasised by the children. |
| Cipolletta, Marchesin & Benini [95] | 2015  Italy | Primary - Qualitative | To explore how family functioning can contribute to the development, maintenance and progression of CI in children. | Documentary analysis of clinical files and semi-structured interviews with 33 parents of children hospitalised in a palliative care unit and the doctor in charge. The data was statistically analysed. | - The demands of coping with a CI create barriers to healthy family functioning, particularly in the case of uncertainty about the diagnosis, focus on or resistance to the illness, reduction in social relationships and inability of the carer to rest.  - The factors that facilitate family functioning reported are certainty of diagnosis, confidence in their children's potential for independence, flexible working arrangements, the presence of siblings, sharing with other families, spirituality and family support.  - Depending on family functioning and its conditioning factors, positive response patterns are generated - flexibility and adaptability, relief, acceptance of the child's state of health and autonomy, maintenance of social roles - and/or negative ones - fear, withdrawal from professional activity, marital and family problems, denial, overload and revolt. |
| Collins et al. [64] | 2016  Australia | Primary - Qualitative | To learn about the experiences of parents caring for children with LLI | This is a cross-sectional, prospective study carried out using semi-structured interviews with 14 parents, whose contributions were subjected to thematic analysis. | - The lived experience of parents of children with LLI/LTI shows that there is a huge need for physical and emotional support beyond what is currently offered in PPC.  - A number of factors, such as physical and social isolation, negative stigma associated with their child's illness, unemployment, and social pressure to assume responsibility and caregiving duties, have been reported to hinder the transition processes experienced by these parents. These factors, together with the uncertainty associated with the progression of the disease, often have an impact on the parents' physical and psychological well-being.  - A greater availability of psychological support services, the existence of resources for home healthcare and public policies that increase accessibility to this care facilitate the transitional process. The operationalisation of strategies such as the definition of routines and the use of equipment generates particularly positive results for children. |
| Connor, Downing & Marston [54] | 2017  USA | Primary - Quantitative | Estimate the global and exact need for PPC. | Cross-sectional study, based on the analysis of primary and secondary data collected from a representative sample of countries from all regions of the world and all World Bank income groups. | - The poor implementation of PPC makes it difficult for children with LLI/LTI illnesses to access it.  - It is estimated that between 20 and 120 children per 10,000 may need PPC, totalling around eight million children globally.  - Estimating the need for PPC at a global level is an essential step towards meeting the needs of children with LLI/LTI, and provides an opportunity to advocate for its expansion. |
| Cotê, Payot & Gaucher [123] | 2019  Canada | Primary - Qualitative | To explore the challenges associated with providing PPC in the emergency department (ED); to describe the potential roles of the ED in providing PPC from the perspective of the services involved in CMC care; to propose concrete solutions to better implement PPC in the ED. | Semi-structured interviews were carried out with six groups from different areas involved in the study - PPC, complex care, PICU and paediatric ED. Each group included between 8 and 10 professionals. The results were subjected to thematic analysis and triangulation. | - Given the high number of children with palliative care needs, care needs to be redesigned to meet these needs, prioritising communication with the family and professionals who know the case, respecting the defined goals of care.  - The provision of PPC in the paediatric ED is seen as a solution that promotes this reorganisation, with the potential to generate positive responses in parents and children, such as satisfaction with individualised care, feelings of support and symptom control.  - Barriers to implementing this approach persist: in the ED, due to lack of training and inadequate space; in the external context, due to lack of continuity of care, stigma surrounding PPC and hospital organisation. |
| Deming et al. [50] | 2022  USA | Primary - Quantitative | To describe the intensity of care and primary and secondary PPC provided to chronically ill children (CIC) at the time of and one year after discharge from the Neonatal Intensive Care Unit. | A multicentre retrospective study analysing the clinical files of technology-dependent CIC discharged after 42 weeks of corrected age. The data was statistically analysed. | - The children who benefited from PPC were those with a diagnosis of CI who had needs in terms of family support, discussion about the goals of care, decision-making, symptom management, communication and coordination of care.  - Follow-up was carried out during hospitalisation, and they were often no longer regularly monitored by the PPC teams after discharge.  - The provision of PPC in neonatology and with continuity after one year is an intervention with the potential to support the transition between hospital and home, generating a sense of long-term support perceived by parents. |
| Desai et al. [113] | 2016  USA | Primary - Qualitative | To explore the needs and preferences of caregivers for the pursuit of high-quality transitions in children between hospital and home; and to describe similarities and differences in the needs and preferences of caregivers according to the CMC. | A qualitative study carried out through interviews with 18 carers of children and adolescents, with an analysis methodology based on Grounded Theory. | - The transition between hospital and home is one of the transitional processes experienced by CIC and their parents, and is associated with important needs on the part of carers to improve the quality of care.  - Social isolation, as well as the uncertainty related to the chronic nature of the disease and its prognosis, are factors that potentially hinder this transition.  - The philosophy of family-centred care, as well as the existence of adequate family, community, school and hospital support, and participation in mutual aid groups were identified as aspects that can facilitate the transition process.  - The perspective of these carers gave rise to a multidimensional theoretical model, focusing on the carer's self-efficacy for care management as a central construct. Within this framework, professionals are called upon to prepare parents in the discharge process, empowering them and providing them with comprehensive written indications about care needs and therapeutic regime. |
| Di Riso et al. [65] | 2020  Italy | Primary - Quantitative | Evaluating play, coping strategies and psychological symptoms in children with CI | Questionnaire including scales aimed at assessing the phenomena of interest, applied to 44 school-age children with CCC. The data was statistically analysed. | - In children with CIs, the existence of a positive affective tone and emotional climate associated with play generates a set of positive feelings.  - Specifically, make-believe play provides a variety of positive characteristics, such as optimising coping with stressful events, well-being, improving emotional expression, adapting to illness and optimising interpersonal relationships. |
| Dunbar, Carter & Brown [66] | 2020  United Kingdom | Primary - Qualitative | To explore the perspectives and experiences of parents of children with LLI admitted to a palliative care unit; to understand the barriers and facilitators to accessing a palliative care unit; to understand the characteristics outlined by parents for the provision of PPC | Focus groups with 24 parents of children with LLI hospitalised in a palliative care unit and, at a later stage, semi-structured interviews with 7 parents who had not used this unit and 7 parents with previous experience of using it. | - Providing PPC generates different results for parents of children with LLI. Trust in health professionals, security with the care provided and familiarity with the ideology of PPC are all factors that facilitate its implementation. Positive results are associated with self-confidence, insideness, optimisation of socialisation and normalisation of daily care.  - This care can be hindered by the notion of uncertainty about the future and evolution of the illness, as well as the social exposure associated with identifying the palliative care unit as a place of death. The length of time required for this type of care is an obstacle to its provision by parents, who have few opportunities to give the carer a break. These factors can lead to negative reactions such as overwork, anxiety, alienation, guilt, fear and reduced socialisation. |
| Fernandes [68] | 2018  Portugal | Primary - Quantitative | To examine the relationship between the family context and adaptation to paediatric cancer; to analyse whether quality of life, rituals, cohesion and hope vary according to the socio-economic level and professional situation of the carers. | Questionnaires containing assessment instruments related to family rituals, family cohesion, anxiety, hope and quality of life were administered to a sample of 90 children and adolescents with cancer, aged between 7 and 20. The data was statistically analysed. | - The family context is more cohesive in the presence of factors that facilitate the family's adaptation to paediatric cancer and its quality of life, namely the presence of rituals, high levels of hope and reduced anxiety.  - Parental unemployment is a socio-economic barrier to adapting to the health condition. |
| Fernandes et al. [67] | 2015  Portugal | Primary - Quantitative | Validating the translation and cultural adaptation of the Adolescent Paediatric Pain Tool in Portuguese children with cancer | Independent process of translation and back-translation of the instrument, analysed by reviewers until consensus was reached and semantic validation by the adolescents was involved. | - The Adolescent Paediatric Pain Tool is used as a pain assessment tool for hospitalised children in multiple contexts, including PPC.  - This tool has innovative and dynamic features, including a body diagram for locating pain and a scale of verbal descriptors for assessing pain intensity, with an open space for personalising the description of the painful experience. The present study materialises its validation for the Portuguese population.  - The use of this scale will help to optimise the symptomatic control of children with CCC, as well as the inherent cultural congruence. |
| Fernandez et al. [96] | 2019  Brazil | Systematic Literature Review | To systematise a definition of decision-making in the provision of care to children with CI in a universe of negotiation between families and health professionals, addressing the specificities and challenges associated with its operationalisation. | Systematic review and thematic analysis of the literature by searching for evidence in the Pubmed and Lilacs databases. Thirty-three articles were selected for subsequent thematic analysis using Bardin's theory. | - Decision-making is a very important aspect of health care and is a process that takes place in the therapeutic relationship between health professionals and the child and family.  - Highly complex situations, such as those involving discussions about the end of life, can be a major obstacle to decision-making.  - A decision-making process is always the result of each person's experiences and is not disconnected from their beliefs, the meanings they attach to the illness, the way they manage their limitations and how they share these decisions with their surroundings.  - Shared decision making as a construct of family-centred care facilitates transition processes, provided that the decision is based on the child's and family's choice, with the professional adopting a consultative and educational stance. |
| Ferreira [125] | 2021  Portugal | Narrative Literature Review | Analysing the experiences of parents whose children have been admitted in PPC units | Search on Google Scholar, Scielo, RCAAP, Institutional Repository of Fernando Pessoa University and B-On. Eight articles were included. | - In relation to the experience of being a mother or father of a child with palliative needs, there are positive responses to being in a PPC unit, such as satisfaction with care, and more negative ones, including fear of loss, sadness, and work/professional problems. The diagnosis of cancer is the main obstacle to this experience.  - The work and emotional support provided by the nurses is considered to facilitate a positive experience in the unit. |
| Fonseca [69] | 2015  Portugal | Primary - Qualitative | Understanding the influence of therapeutic letters on the hopes of parents of children with CI | Focus group with 15 nurses to draw up therapeutic letters and semi-structured interview with 10 parents of children with CI | - The promotion of hope is a facilitator of parental experience of their children's CIs, as well as support, availability and recognition of parental competences by professionals.  - Therapeutic letters provide an opportunity to share hope with health care professionals in practice. This strategy has the potential to strengthen the therapeutic relationship and promote parental hope.  - The implementation of the therapeutic letters has strengthened the parents' personal strength in the face of the adversities they have.overcome. |
| Foster et al. [134] | 2017  USA | Primary - Qualitative | To describe the perspective of health professionals and hospital administrators on how to improve the transitions of children and young people with CI between hospital and home. | Focus groups and semi-structured interviews were carried out with the participation of 22 key informants in the clinical context. The data was then coded. | - High-quality transition between hospital and home for children with CI requires structure (multidisciplinary team for continuity of care, with guaranteed availability of resources in the community) and defined processes (individualised goals, involvement and training of parents in care and regular communication with primary health care). The lack of consistency in these criteria leads to the need to improve the quality of these approaches.  - Optimisation of psychosocial and family functioning, improved management of the therapeutic regime and a reduction in the number of readmissions were identified as positive responses to high-quality transitions. |
| Fraser et al. [55] | 2021  United Kingdom | Primary - Quantitative | Estimate the current prevalence of children with LLI and model the future prevalence of this population | Observational study using clinical data from a hospital. A population-based methodology was used to estimate future prevalence. | - The prevalence of children with LLI is increasing and is approximately 66 per 10,000 live births, with the majority of cases occurring in children under 1 year of age and those with congenital malformations.  - The assessment of the need for palliative care should be carried out at all ages in the presence of an LLI, with special attention to the first year of life. |
| Galligan et al. [48] | 2018  USA | Case Study / Project | Proposing a tertiary care medical centre model based on the experience of the Pediatric Aerodigestive Centre | Description of the intervention methodology at the Paediatric Aerodigestive Centre, based on a clinical case of a child with complex health needs. | - CMC have complex health needs, being affected by multi-systemic diseases, requiring polymedication and frequent follow-up by specialists, making the coordination of care at hospital and community level critical. They can be technology-dependent.  - The existence of a tertiary medical centre can improve the multidisciplinary monitoring of these children in the community and optimise the coordination of care. The presence of these centres is seen as a positive response to transition, optimising the wellbeing of the child and family. |
| Gien et al. [135] | 2017  USA | Primary - Quantitative | Evaluating the impact of an interdisciplinary ventilator-associated care programme for children with bronchopulmonary dysplasia | This retrospective study analysed the clinical files of children with this pathology. The data collected was statistically analysed. | - The implementation of an interdisciplinary ventilator care programme, made up of a multidisciplinary team with collaborative discussion and case management methodology, is important in personalising and adapting the child's intervention plan.  - The increase in survival at the time of discharge is a positive response resulting from the application of this programme. |
| Góes & Cabral [120] | 2017  Brazil | Primary - Qualitative | To understand the discursive and social practices of health professionals and family carers regarding the discharge from hospital of children with special health needs. | Qualitative research using semi-structured interviews with six health professionals and eleven family carers. The texts were submitted to critical discourse analysis. | - Three types of discharge are identified - clinical-administrative, procedural and social - in which the roles of each professional are fragmented.  - The operationalisation of an interdisciplinary clinical discharge allows for continuity of care at home with autonomy, safety and quality. |
| Gonçalves et al. [131] | 2022  Portugal | Primary - Quantitative | Assessing the sleep quality of carers of children with CCC on mechanical ventilation at home | This was an observational study using a sleep quality assessment tool with a sample of 33 carers. | - Decreased quality of sleep for parents of children on mechanical ventilation at home is very common, particularly when it comes to children with respiratory diseases, LLI or terminal illnesses.  - In the face of this increase, there are negative responses, such as the risk of parents jeopardising the quality of childcare. |
| Govender et al. [70] | 2015  USA | Narrative Literature Review | To synthesise clinical strategies for empowering children with cancer and developing a fighting spirit towards their physical and mental health | Review of the evidence on interventions to promote empowerment in children with cancer, emphasising the role of the clinic and research into the use of video games. Methodology not specified. | - In the face of an oncological disease, the promotion of empowerment and the use of video games based on concepts of hope and stress reduction can facilitate the adoption of a positive attitude towards the disease.  - Promoting empowerment is therefore an important intervention for improving the clinical condition of children with cancer. It can be implemented through the traditional method (through therapeutic education, conferences, meetings with other children with cancer, group sessions and exercise programmes) or the contemporary one (e.g. using technological solutions).  - Physical and mental well-being, as well as improved management of the therapeutic regime, are perceived as the main positive responses resulting from this intervention. |
| Hamner et al. [71] | 2015  USA | Primary - Quantitative | Examining the role of chronic parental stress in explaining the quality of life of children with cancer | Cross-sectional descriptive study using a questionnaire to measure the time since diagnosis, the child's quality of life and their chronic stress | - Chronic parental stress is a major barrier to the quality of life of children with cancer and their parents.  - The presence of this type of stress generates negative responses, such as physical and mental malaise, as well as reduced socialisation skills in children with cancer. |
| Hill et al. [52] | 2014  USA | Primary - Qualitative | Presenting a conceptual model for the transition of goals in parental decision-making about their children's care plans (Regoalling). | Literature research on factors relevant to the Regoalling process, integrating them into a conceptual model to be tested. The implications of this model for PPC practice are also addressed. | - Faced with the diagnosis of a CI, the change in parental objectives when making decisions about the care to be given to their children is part of the transitional process that parents experience.  - Health situations that are not favourable for the child become factors that can hinder parental decision-making, not promoting the reformulation of objectives and keeping them out of step with the existing context.  - Regoalling is a conceptual model that reflects a psychosocial approach designed to support parents in this process of converting goals and expectations, focusing on: respecting each parent's time in the process; preparing parents in advance for the decision, providing them with all the information in a clear and truthful manner; providing emotional support to parents, recognising their qualities in caring for their children; and promoting hope.  - The existence of this "crisis" and the clinical implementation of this model has the potential to improve the quality of life for children and their parents. |
| Hirschfeld et al. [142] | 2019  USA | Primary - Mixed Methods | Identify the adequacy of communication practices within the hospital healthcare team responsible for CMC care, identifying priority areas for improvement | Questionnaire applied to 304 professionals, containing closed questions, where statistical analysis was applied, and open questions, where content analysis was conducted. | - The adoption of appropriate relational practices within the team, based on communication tools and applied in dynamics of collaboration and continuity of care, is fundamental in caring for children with CCC.  - The absence of adequate relational practices can lead to a breakdown in shared decision-making. |
| Kars et al. [88] | 2015  The Netherlands | Primary - Qualitative | To describe and explain the actions taken by parents to express and manage the wishes of a child with incurable cancer in palliative care. | A multicentre qualitative study carried out by interviewing 34 parents of children with incurable cancer in palliative care, with a thematic analysis of the contributions made. | - Expressing and managing the wishes of children with incurable cancer in palliative care is, from the parents' perspective, closely linked to listening to their voice. To this end, direct strategies are described - asking the child directly, assessing verbal and non-verbal cues, statements made in the past and information provided by third parties - and indirect strategies - what the child has said about their feelings, needs, desires or perceptions.  - Parents' denial of the clinical situation of a child with this diagnosis does not favour the opportunity for them to actively participate in decision-making about their condition, avoiding the inevitability of loss and death. Taking the child's wishes and voice into account when planning and managing care facilitates this transactional process. |
| Kuo et al. [128] | 2014  USA | Primary - Quantitative | To determine the association between variables known to be associated with inequities in healthcare needs and the state of medical complexity of children and young people with SHN. | Secondary analysis of the national survey of children with SHN, focussing on variables associated with health inequities. | - The complexity of illness in children and young people with SHN is seen as a primary determinant of inequality, in addition to the traditional determinants which, in this study, did not generate as many inequalities (e.g. hunger, socio-economic status, poverty, ethnicity, etc.).  - The lack of public policies that support the dynamics needed for the diagnosis and management of these CCC hinders the transition that these children and families experience, highlighting the need to adapt legislative instruments to these needs. |
| Lafrenaye et al. [109] | 2021  Canada | Primary - Qualitative | To understand the reasons for differences in the interactions between parents of children with CCC and health professionals | Interview applied to 8 parents of children with CCC in a paediatric consultation. | - The differences in interactions between parents and professionals are linked to the personal and relational characteristics of both parties.  - The establishment of a common spiritual identity is seen as a facilitator of this relational process, generating serenity in the parents.  - Delegation, power, handing over care to third parties and the lack of parental involvement in care are barriers to positive interaction. |
| Leite Colesante et al. [110] | 2015  Brazil | Primary - Qualitative | Understanding the impact of a child's CI on the life of the mother carer | Semi-structured interview with 5 mothers of children with CCC when their children were hospitalised in a public hospital | - The CI of a child is an event whose adjustment is complicated by feelings of fear of the pathology, denial of the diagnosis, guilt, unemployment and socio-economic difficulties.  - Controlling and adapting to these conditions is facilitated by spirituality, which acts as a protective factor for inner balance. |
| Lerret et al. [97] | 2015  USA | Primary - Mixed Methods | Investigating the factors associated with the transition from hospital to home and with chronic illness care | Semi-structured interviews and questionnaires applied to 51 parents from 5 paediatric transplant centres. | - Early discharge planning and family empowerment are crucial dynamics in the safe transition of children home, in a process where the needs of children and their families must be considered.  - Inability to operationalise care is seen as a factor that hinders this transition and can lead to a decrease in self-efficacy at home. |
| Lerret et al. [114] | 2017  USA | Primary - Qualitative | To explore parents' experiences of the transition from hospital to home and the management of CCCs in children undergoing organ transplantation. | Interviews were carried out with 48 parents, the results of which were subjected to content analysis. | - Promoting the empowerment of families to care for children with CCC is an important intervention to guarantee their health, and the family should be involved in managing the illness and advocating for the adoption of healthy lifestyles.  - Ongoing support for parents to understand their role and the experiences and challenges they face in caring for their children, as well as early training and family assessment, will facilitate this transition.  - The adoption of early training and family support strategies by professionals has the potential to generate positive responses, such as effective management of the therapeutic regime and well-being for the child and family. |
| Leyenaar et al. [89] | 2017  USA | Primary - Qualitative | To examine parents' preferences, priorities and goals in relation to planning transitions from hospital to home; and to verify healthcare providers' perceptions of families' transition goals and care needs. | Semi-structured interviews with an intentional sample of parents of hospitalised children and professionals. The content of the interviews was subjected to data triangulation and validation by experts to create a conceptual model. | - Faced with the transition between the hospital and the home of a child who has been hospitalised, the discussion between professionals, children and parents about the need for continuity of care is a challenge.  - The perspective of the parents and professionals culminated in the following priorities for facilitating the transitional process: involving the family in discharge planning; respecting families' preparation for discharge; coordinating care prior to discharge; carrying out timely, efficient and comprehensive discharge processes to provide symptomatic control, self-efficacy to support recovery and adequate development; support in families' daily routines, during and after hospitalisation, and in normalising the illness. |
| Ling et al. [90] | 2016  Ireland | Primary - Qualitative | To analyse the opinions and experiences of parents of children with LLI who are receiving temporary care outside the home (caregiver respite) and to present a new proposal for a care model based on the results of this research. | Semi-structured interviews were carried out with 19 parents, the results of which were later subjected to content analysis. Participant observation, document analysis and field notes were also used. | - The existence of the possibility of respite for the caregiver in PPC is beneficial for the child, but especially for the parents, who have the opportunity during this period to restructure their self and socialisation habits for greater socio-emotional stability.  - When looking for institutions for respite care, parents value respect for the child and their safety, as well as proximity to home, the consistency of the team and appreciation of the child's routines and rituals. Discriminatory attitudes, as well as greater exposure to the risk of infection, are factors that hinder this approach. |
| Looman et al. [98] | 2013  USA | Case Study / Project | Describe advanced nursing knowledge and practice for relationship-based care coordination in homes housing children with complex SHN. | Exploratory analysis of the scope of practice of advanced practice nurses based on the description of the TeleFamilies project | - The commitment to advanced nursing practice is important for improving the coordination of specialised care, particularly family-centred care.  - The existence of a specialised nurse is important to reduce fragmentation and improve the efficiency of the care provided by the health team. Family conferences are one of the most effective working dynamics for this purpose.  - The use of telehealth tools in this process is crucial, as they make it possible to monitor and provide continuity of care for the child and family from a distance. |
| Looman et al. [72] | 2015  USA | Primary - Quantitative | Evaluate the effect of a telehealth intervention promoting care coordination implemented by nurses for CMC on family carers' perceptions of health care | A randomised controlled trial involving a sample of 148 family members, in which satisfaction with healthcare was assessed. | - A telehealth intervention focused on teleconsultation to promote care coordination, as a complementary strategy to home care, is an important resource for articulating care with the community and for the success of health education dynamics.  - The lack of assistance available in the community can make it difficult to implement the intervention and achieve positive responses to the transition. |
| Lopes [53] | 2016  Portugal | Primary - Qualitative | To find out what information/training needs parents consider important in order to continue caring for their child after hospital discharge | A descriptive and exploratory study, carried out through documentary analysis and interviews with three mothers of children with special health needs. | - In the process of training for discharge, parents value a global informative approach to the child's needs, particularly in terms of self-care, emotional management, carrying out specific procedures, social support and training.  - Faced with a new CI, there are various factors that hinder the empowerment process, such as lack of parental experience, fear, anguish, stress and socio-economic difficulties.  - Adopting a strategy of early preparation for discharge, based on a supportive and available relationship between staff and parents and in-depth knowledge of the family and its social context, can reduce uncertainty and fear of the unknown. |
| Lopes [73] | 2019  Portugal | Primary - Qualitative | To analyse which nursing interventions are carried out during consultations with family members and children with DM1 in order to promote adherence to the therapeutic regime; to find out the main difficulties these professionals experience when carrying out the consultation; and what the main feelings expressed by the child and family are in the face of the diagnosis. | An exploratory and descriptive study, carried out using semi-structured interviews with 9 nurses who provide diabetes consultations to children and young people. | - The main interventions carried out in this consultation are related to the definition of therapeutic plans with the involvement of the child and parents, focused on promoting knowledge about the disease and skills for managing the therapeutic regime. These dynamics are hampered by the short time available for consultations, insufficient ratios and lack of privacy.  - Aspects that facilitate the process of adherence to the therapeutic regime and acceptance of the disease are identified, namely: seeing the family as partners in care, as well as the use of non-pharmacological and interactive strategies, such as videos and therapeutic games. Therapeutic communication and positive reinforcement are also facilitating factors. |
| Lotz et al. [74] | 2017  Germany | Primary - Qualitative | To investigate parents' perceptions and needs in relation to advanced care planning in paediatrics. | Semi-structured interview with a sample of eleven parents of children with different conditions and contexts of care. | - Open, honest, transparent communication that informs shared decision-making is essential for family-centred care partnerships.  - Gradual dialogue focused on the child's needs between the parents and the multidisciplinary team is a factor that facilitates advanced care planning, and addressing hope and psychosocial issues beyond the illness is seen as a necessity.  - Involving parents and children in decision-making, assigning a reference nurse, and providing materials that optimise self-determination and hope are interventions that have been identified as having the potential to reduce negative feelings, such as uncertainty and fear, and improve the safety and quality of the care provided. |
| Macaulay et al. [115] | 2020  New Zealand | Primary - Qualitative | To identify the factors related to DM1 that affect, as well as the solutions that improve, the sleep of parents of children with DM1 | Semi-structured interviews with 10 fathers and 10 mothers of children with DM1, in which the Pittsburgh Sleep Quality Index was applied and sleep was assessed using actigraphy. | - The sleep of parents of children with DM1 is positively affected by the use of cost-effective technological solutions that reduce the fear associated with changes in their child's glycaemic pattern and negative feelings when falling asleep.  - The use of alarm systems connected to capillary blood glucose assessment devices, as well as continuous blood glucose monitoring and percutaneous insulin administration are all solutions that make it easier for parents to improve their sleep. Other solutions include support from family and third parties, as well as shared night-time care between carers. |
| Magão [75] | 2017  Portugal | Primary - Qualitative | Understanding the lived experience of hope for parents of children with a CI | A study with a phenomenological and interpretative design, operationalised through interviews with 7 parents of children with CI. | - Hope is an emotion with therapeutic potential, particularly in times of crisis. In this sense, feelings of uncertainty, adversity and loss can be addressed through hope-promoting strategies.  - These strategies are implemented through mutual help groups between professionals and parents of children with chronic illnesses, as well as by building narratives of hope in communication with parents and in the therapeutic relationship. |
| Martins [132] | 2019  Portugal | Primary - Quantitative | To assess the quality of sleep and quality of life of carers of children/adolescents with CCC and the need for home mechanical ventilation, relating them to sociodemographic and clinical characteristics. | A prospective, exploratory and observational study was carried out using questionnaires administered to 36 carers, assessing quality of life and sleep quality. | - Parents of children with CCC develop disruptive coping mechanisms when they experience sleep disturbances, such as sadness, despair, anxiety and depression. These negative response patterns can be aggravated in the event of overload, which is found to be more common in parents of children with high technological dependency.  - The existence of alterations in the sleep of these children's parents should be seen by health teams as a warning factor, and it is important to anticipate strategies to reinforce family support and psychological intervention, if necessary. |
| Mavis et al. [76] | 2015  USA | Primary - Quantitative | To identify the patterns of child and family adaptation among kidney and liver transplant recipients in relation to vulnerability, the impact of the disease on the family, family functioning and quality of life. | A cross-sectional study was carried out using questionnaires administered to 47 children and their parents. Subsequent statistical analyses were carried out. | - Performing a kidney and liver transplant on a child implies a vulnerability associated with the health condition, which, in the opinion of the parents, has the potential to diminish the quality of life of both the parents and the child.  - Psychosocial support is a factor that can positively condition this process. |
| McKissick et al. [121] | USA  2017 | Primary - Mixed Methods | To evaluate the effects of a telehealth intervention practised by nurses on the use of health services by CMC | A randomised controlled trial involving two intervention groups. | - The implementation of a telehealth intervention practised by nurses has the potential to reduce unplanned hospital visits, which would be higher in number due to the complexity of CCC. |
| Mellblom et al. [130] | 2015  Norway | Primary - Qualitative | To find out whether the topic of the late effects of antineoplastic therapy is discussed in consultations with children who have survived cancer, and to identify whether this information was passed on by paediatric oncologists. | This study was carried out by recording consultations with 10 paediatric oncologists and included 66 adolescents. When approached, the data relating to this topic was coded. | - Passing on information about the medication regime, particularly about its adverse physical and psychosocial effects, is a topic that parents value in the training process.  - Failure to communicate this information to parents has the potential to reduce their ability to manage the therapeutic regime, increasing the risk associated with the use of the medicine. |
| Melo [77] | 2018  Portugal | Primary - Mixed Methods | To analyse the effects of Doctor Clowns in promoting the well-being of children/adolescents with cancer during chemotherapy. | Study with a mixed design - quantitative experimental and qualitative exploratory approach. Methodology operationalised through three studies aimed at the needs of these children, the contributions of Doctor Clowns intervention in the management of the disease and treatment, and the effects of this same intervention. | - The intervention of the Doctor Clowns focuses on activities that promote emotional expression, abstraction, education, occupation and socialisation for children, seeking to increase their well-being.  - This intervention creates a positive emotional climate, allowing for a less negative memory of the experience. |
| Monteiro [78] | 2019  Portugal | Primary - Mixed Methods | To explore parental perceptions of the experience of paediatric cancer, as a couple and as parents, including the possible changes resulting from the experience of the disease process. | A mixed design study, operationalised through a questionnaire applied to 48 parents of children and young cancer survivors, in which parental experience and psychopathological symptoms were assessed, and a semi-structured interview with 27 of these parents. | - Faced with a paediatric cancer illness, the parents of a sick child can develop negative response patterns, such as anxiety about the child's hospitalisation and pathological process, confrontation with the reality of other children, sadness, depression, anger and guilt. These responses can be aggravated by a lack of social support and overload.  - The implementation of a therapeutic relationship that promotes hope, supporting parents in an empathetic, available and close way, as well as transmitting the necessary information with respect for their perceptions and choices, is a factor that facilitates this experience, with the potential to provide relief and tranquillity. |
| Mooney-Doyle et al. [99] | 2018  USA | Primary - Mixed Methods | To describe the challenges faced by parents in prioritising and differentiating tasks, as well as the appropriate parenting style for children with LLI with healthy siblings. | Semi-structured interviews focusing on the management of children's needs and questionnaires focusing on the prioritisation of tasks between the child with a LLI and the healthy sibling were administered to parents of children with these illnesses. | - Parents say that caring for the needs of a child with a LLI is related to managing expectations, differentiating roles - essential support for clinical needs versus relational support for managing communication between siblings at home - and individual, couple and family burdens. Showing affection and focusing on the children's health is an attribute of good parenting for both siblings.  - In the case of the healthy sibling, the parents ensure an effective role by focusing on quality of life, comfort and spiritual well-being. However, for the child with a LLI, the priorities change: the focus is on clinical decision-making, the role of advocate towards health professionals and parental presence, which can facilitate the transition and management of the impact of these illnesses on the household.  - Understanding the multiple sources of parental stress and their role in managing the LLI at home and in the family is an intervention that can help generate support infrastructures and avoid negative effects on the family. |
| Mororó et al. [79] | 2020  Brazil | Primary - Qualitative | Understand the nurse's practice in managing care for children with chronic conditions in a hospital setting | An ethnographic study, carried out through participant observation, documentary analysis and semi-structured interviews with 20 nurses, | - In a hospital setting, the nurse assumes the duty of partnering with the family in caring for the child, trying to enable them to become autonomous and empowered for the continuity of care after discharge. They are also responsible for making referrals and liaising with professionals in the community.  - The mediation and partnership attitude adopted by the nurse in the management of care, as well as the positive nature of the interpersonal relationships established within the healthcare team, facilitates the provision of care. |
| Murphy & Ehritz [80] | 2021  USA | Case Study / Project | To highlight the nurse's practice in identifying complex CMC care needs, describing the resources needed for a successful transition between care settings and effective continuity. | Collecting data with unspecified methodology on a child with epidermolysis bullosa and searching for scientific evidence on appropriate interventions. | - Identifying children's palliative needs should be done through individualised and detailed assessment and care planning, based on the best scientific evidence. The high workload and overload of nurses make it difficult to realise this strategy.  - Communication, as well as the creation of teams of reference nurses and individualised daily care documents specific to each child, are strategies that facilitate continuity of care and facilitate effective transition between settings. |
| Muscara et al. [143] | Australia  2020 | Primary - Quantitative | To evaluate the effectiveness of a group intervention focused on acceptance and commitment, carried out via videoconferencing, in reducing symptoms of post-traumatic stress in parents of children with LLI. | This is a randomised controlled trial, conducted through the construction of an intervention group. | - The use of acceptance and commitment therapy, aimed at parents of children with a LLI, has led to important health gains for this population, particularly in terms of stress reduction.  - The aggravation or emergence of post-traumatic stress disorder emerged as a possible negative response pattern. |
| Nageswaran et al. [100] | 2014  USA | Case Study / Project | Reflect on the limitations and strategies associated with the transition process between hospital and home in children with LLI | Data collection with unspecified methodology on cases of transition between hospital and home for children with LLI. | - The process of transitioning a child with a life-limiting illness between hospital and home can be hampered by communication barriers within the interdisciplinary team, as well as a lack of time on the part of professionals.  - The use of approaches such as discharge checklists, therapeutic reconciliation, interdisciplinary meetings, discharge planning, post-discharge follow-up and the existence of support services are important to support the child and family in this transition. |
| Nightingale et al. [91] | 2015  United Kingdom | Systematic Literature Review | To identify parental needs and preferences for managing the long-term needs of children with CI, helping to identify interventions by professionals in this regard. | Systematic literature review using seven databases from 1990-2013. The quality of the studies was assessed. | - Parents identify as facilitators the adoption of information adapted to the individuality of each child and family, as well as the assignment of health professionals with better teaching, instruction, training and therapeutic relationship skills.  - On the other hand, professionals with fewer teaching skills, limited time and resources and lack of information about parental needs and preferences are factors identified as barriers to managing these transition processes.  - The adoption of therapeutic interventions in partnership, based on real parental needs and perceptions, is essential to optimise the provision of health care to the child and family, improving the resulting health gains. |
| Nolte-Buchholtz, Zernikow & Wager [108] | 2018  Germany | Primary - Quantitative | To describe the demographic and clinical characteristics of children referred for specialised paediatric palliative home care, comparing the situation of children with oncological and non-oncological conditions. | A longitudinal (cohort) and prospective study, operationalised through a questionnaire applied to the situation of 75 children with palliative needs. | - Children in PPC had a wide range of conditions, from neurological compromise, pain, compromised communication or swallowing and seizures. The use of devices such as nasogastric tubes, as well as oxygen, tracheostomies and ventilation was used in the vast majority of patients.  - Given the complexity of the situations described, a PPC team made up of highly qualified professionals with multiple skills is a strategy that facilitates its application and implementation. |
| Noyes et al. [101] | 2013  United Kingdom | Primary - Mixed Methods | Describe the development, implementation and evaluation of innovative palliative care planning resources led by children and parents. | Initial literature review, production of planning resources (My Choices booklets) and evaluation through interviews and questionnaires. | - The booklets produced were seen as a tool that could help to foresee the future of care, in terms of the choices and contexts in which it is provided, as well as discussion between families and health professionals. Their use varied, but they were identified as facilitating the recording of information and the weighing up of therapeutic options.  - The use of the booklets improved awareness of care planning and promoted the organisation of parents' thinking about their children's care needs, the context of care, partnership in this process and bereavement. |
| Nikfarid et al. [144] | 2015  Iran | Primary - Quantitative | Investigating chronic sadness in mothers of children with cancer in the hospital environment | A descriptive, cross-sectional study was carried out by applying a questionnaire to a sample of 264 mothers and then analysing the data statistically. | - Facing a situation where your child is ill and hospitalised can lead to a feeling of reduced self-efficacy, which can be aggravated by poor family and social support, as well as chronic sadness. |
| Oakley et al. [81] | 2022  United Kingdom | Systematic Literature Review | Identify strategies initiated by parents of children with LLI to support their own well-being at home and describe the impact of these strategies on parental well-being | The study used a scoping review design, in accordance with the PRISMA guidelines, culminating in the identification of 15 articles. | - Faced with the need to improve the well-being of parents of children with LLI, the existence of peer support and the sharing of responsibilities between them, as well as the prioritisation of needs and belief in the role of spirituality have the potential to facilitate this process.  - Decreased socialising and social isolation make it difficult to maintain well-being at home. |
| Palma et al. [49] | 2015  USA | Primary – Mixed Methods | To examine the daily maternal care needs of adolescent and young survivors of  brain tumours who live with their families. | This was an exploratory study, carried out using semi-structured interviews with 46 mothers of adolescents and young survivors of cancer. | - Adolescents and young people who have undergone brain tumours do not fail to present various needs, particularly related to self-care and functionality. These needs can worsen in the presence of parental stress and emotional distress.  - The process of empowerment, through teaching dynamics, instruction and training on managing the therapeutic regime and emotional control, is fundamental to safeguarding these daily needs. The existence of a parental support network at a social and psychological level can facilitate this process. |
| Pelentsov et al. [111] | 2013  Australia | Primary – Mixed Methods | Identifying the supportive care needs of parents with a child with a rare disease | A study with a mixed design – focus groups and an online questionnaire. | - Parental care for a child with a rare disease requires a lot of time and commitment, which can be negatively affected by anguish, despair and fear of neglect by other siblings. The persistence of these feelings can lead to social isolation, denial of the health condition and family dysfunction.  - The existence of support groups for parental communication, carer respite, the promotion of spirituality and psychological counselling are an asset in identifying and managing these needs. |
| Palaré et al. [56] | 2023  Portugal | Primary – Quantitative | Translate, culturally adapt and validate the PaPaS scale for the Portuguese paediatric population | Quantitative methodological study of translation, cultural adaptation and validation, with evaluation of the instrument’s psychometric properties. | - Palliative needs are mostly recognised late, which is associated with the limited number of assessment tools available.  - The Paediatric Palliative Screening Scale (PaPaS Scale) was designed to help professionals identify children/young people with a complex, LLI or LTI who would benefit from paediatric palliative care and facilitate timely and appropriate referral. |
| Pereira et al. [102] | 2018  Portugal | Primary - Qualitative | To understand how and what strategies carers of children with CI in paediatric oncology use to reconcile their professional, family and personal lives. | Interviews with 12 parents of children with an oncological disease and documentary analysis. The data was subjected to content analysis. | - Reconciling personal, professional and family life is made more difficult with the diagnosis of a CCC in the child, and family adjustment can decrease, particularly in the early stages of the illness.  - The social support provided by family and informal support networks, as well as the sharing of household tasks and responsibilities and family cohesion facilitate reconciliation. |
| Pimentel et al. [106] | 2017  Brazil | Primary - Qualitative | To understand the perception of parents of children and adolescents when faced with the diagnosis of DM1. | A descriptive and exploratory study with a qualitative approach, carried out using a semi-structured questionnaire and subsequent content analysis. | - The diagnosis of DM1 was identified as an experience that alters the experiences of the child and family, generating negative feelings such as despair, suffering and anxiety.  - The support of health professionals through appropriate training, as well as integration into a community support network, training dynamics on subcutaneous insulin infusions and the assignment of a reference nurse are identified as facilitators for the transition between a healthy life and everyday life with diabetes.  - Parents' overprotection of their children with diabetes has the potential to create difficulties in acquiring autonomy and making decisions about their health. The inherent costs were pointed out as a negative dimension in this process. |
| Ramalho et al. [140] | 2022  Brazil | Primary - Qualitative | Analysing the performance of nurses in discharging children with CI from hospital | Semi-structured interviews with 10 nurses, which were then subjected to thematic analysis. | - Nurses recognise as their duty the early preparation for discharge, from admission onwards, focusing on the knowledge and skills needed to carry out care at home. However, the realisation of these practices is weakened by the shortage of nurses and the lack of coordination between the multi-professional team and the health system in general.  - The barriers to carrying out the appropriate procedures when discharging children with CI from hospital make them more susceptible to negative response patterns, such as a reduction in the well-being and autonomy of children and parents. |
| Rindstedt [93] | 2014  Sweden | Primary - Qualitative | To investigate the various ways used by health professionals and parents to support the coping of children with cancer. | A study using ethnographic methodology operationalised through the analysis of interactions between children, parents and professionals recorded on video. | - Collaborative storytelling, humour in treatment practices, playful rituals, role-playing and improvisation are all aspects that facilitate the child's coping.  - As for parents, the benefits of this approach are centred on increasing involvement in care and taking parents' perceptions into account when operationalising it. |
| Rogers et al. [47] | 2021  USA | Concept Analysis | Conduct a concept analysis on the term "CMC" | Study design based on Walker and Avant's framework for developing concept analysis, exploring its meanings, attributes, antecedents and consequences. | - CMCs are a growing population worldwide, encompassing specific attributes such as the CCC and its multisystem involvement, functional limitation and the need for articulation of care between all the resources that accompany the child and family. They are often dependent on medical technology to support their vital functions.  - The growing prevalence of CMC calls for reflection on the interventions to be implemented, given the need to respond to high-cost transitional dynamics and, at the end of life, the chronic grief of family members.  - Nurses are ideal advocates and carers for these children, so the development of interventions aimed at this population by this professional group has the potential to promote positive response patterns. |
| Romano [133] | 2021  Portugal | Primary - Quantitative | To assess coping and its relationship with various clinical, sociodemographic and psychosocial factors, namely family carer burnout and post-traumatic growth, in carers of children/adolescents with CI. | A quantitative correlational study, carried out using a questionnaire applied to 127 carers. The data was statistically analysed. | - The wear and tear on the carer is a factor that hinders the experience of caring for a child with a CIs.  - Post-traumatic growth is seen as a protective factor in this experience, enhancing the acquisition of coping and family resilience, which in turn are seen as positive response patterns. |
| Schütze et al. [82] | 2022  Germany | Primary - Qualitative | Exploring parents' experiences and requirements when it comes to working with teams specialising in outpatient PPC | Interviews with 13 parents of children with LLI, using the Grounded Theory approach to analyse the data. | - Taking into account the parents' experiences, the professionals' clinical expertise in paediatrics, honesty, open communication, availability, appreciation of psychosocial support and recognition of the importance of an individualised approach are attributes valued by parents when working with these teams.  - Empowerment to manage the therapeutic regime was also pointed out as a facilitator of this collaboration, along with the recognition of parents' expertise by professionals (through involvement in care and respect for their perception).  - The existence of teams with these characteristics has the potential to generate positive response patterns, particularly in terms of relieving the burden, preventing the child from being hospitalised, optimising self-efficacy and self-management, mental well-being and satisfaction with the care provided. |
| Seear et al. [122] | 2016  Canada | Primary - Qualitative | Understand the impact of paediatric home ventilation on families' daily lives. | Questionnaires with questions on demographic information and quality of life, with subsequent telephone monitoring of the social and financial costs of care over 8 weeks. | - From the point of view of the child under ventilation, transporting this technique to the home environment is apparently a successful strategy, since if it weren't possible, a significant number of these children would not survive.  - Home support provided by nurses is mentioned as being of great importance, along with community resources, as it facilitates the process of caring for these children.  - The persistence of barriers to care, such as the lack of free ventilators and the high costs associated with their maintenance, has the potential to generate financial difficulties for the family and increase stress for carers. The social exclusion resulting from this physical limitation is also pointed out as a factor that complicates this process. |
| Sebastião [129] | 2016  Angola | Primary - Mixed Methods | Investigating physiotherapy intervention in infantile cerebral palsy in the city of Luanda | This was an exploratory and descriptive study, carried out by administering questionnaires to 34 physiotherapists and 33 carers. The data was analysed statistically. | - The involvement of physiotherapy professionals in the care of children with cerebral palsy is extremely important, particularly through the implementation of balance training, muscle strengthening and gait training interventions.  - Parents' lack of knowledge about their child's illness, as well as unfavourable socio-economic conditions, can make it difficult for these professionals to intervene in these cases. |
| Silva [116] | 2014  Portugal | Primary - Qualitative | To identify the feelings experienced by parents after their child was diagnosed with DM1; to understand parents' experiences of caring for a diabetic child; to reflect on the influence of the care provided by health professionals on the acceptance and adaptation to diabetes on the part of the child/adolescent and their family. | This is a phenomenological, exploratory and descriptive study, carried out using semi-structured interviews with 14 parents of children or adolescents with type 1 diabetes mellitus. The data was subjected to content analysis. | - At the time of diagnosis and adaptation to DM1, the manifestation of negative feelings such as fear, anger, guilt and sadness, as well as uncertainty about what the future holds in terms of the progression of the disease and its impact on personal, social and family life, has the potential to make it difficult to experience this transition process. Fears about complications from the disease and errors in managing the therapeutic regime can amplify this feeling.  - In this context, the adoption of collaborative approaches within the family, with understanding and help from siblings, and with health professionals, are facilitators of this experience. Improving knowledge about the disease in the school environment and in the community in general, as well as organising training sessions for parents and children with DM1 were also identified in this context.  - The implementation of facilitating strategies leads to positive response patterns, particularly in terms of the flexibility of family dynamics, the development of new skills for adapting to the role of parent and carer, and the contribution to balance and acceptance of the illness. |
| Silva [126] | 2018  Portugal | Primary - Quantitative | To characterise the biopsychosocial risk profile of adolescents with CI followed up in consultations at a paediatric hospital; to assess whether there are differences in the profiles of these adolescents in relation to the population without CI; to assess whether there are biopsychosocial risk profiles among adolescents with CIs that are most associated with difficulty in adhering to therapy. | A retrospective and exploratory cross-sectional study, operationalised by applying a questionnaire to a sample of 197 adolescents, 117 of whom formed the control group and 80 the study group (with DM1 or juvenile idiopathic arthritis). | - As a result of experiencing CI, various biopsychosocial risk factors have been identified in these adolescents, namely frequent and rapid tiredness, sedentary lifestyles, school absenteeism due to the need for clinical follow-up, discrimination, parental overprotection, a demanding therapeutic regime, family dysfunction, parental unemployment and mood swings.  - Risk factors are associated with negative response patterns to chronic illness and the experience of adolescence, in particular the prevalence of secondary health problems, lower school performance and poor adherence to therapy.  - Nevertheless, the majority of adolescents with these CI show positive responses and are well adapted to the needs arising from them. |
| Sousa [103] | 2014  Portugal | Primary - Qualitative | Exploring nurses' therapeutic intentions associated with promoting care partnerships with parents during a child's hospitalisation | An action-research project carried out in a hospital paediatric unit, using observation, questionnaires and interviews with parents and nurses. | - The experience of a child's hospitalisation creates constraints for parents that can make it difficult for nurses to intervene in this context, such as uncertainty about the clinical situation, the child's relocation from home to hospital, the commitment to the child's well-being, the clash of expectations and the change in parental behaviour. The need to acquire new knowledge and skills is also considered a potential barrier.  - The therapeutic intent of nurses when dealing with a child with CHD is thus evident in the various dimensions that respond to these problems, from promoting the participation of parents in care and valuing their perceptions to preparing and encouraging the acquisition of skills to provide complex care.  - The care partnership is a key facilitator in this therapeutic process, with the potential to reduce the parental stress associated with the complexity of the knowledge and skills to be acquired and allowing for flexibility in the provision of care - on the one hand, increasing the autonomy and performance of the parents and, on the other, providing space for the carer to rest if desired. This makes it possible to develop positive response patterns in order to construct new meanings about their lives and those of their children and to optimise the complex parental role. |
| Sterni et al. [136] | 2016  USA | Primary - Qualitative | Develop evidence-based clinical practice guidelines on hospital discharge and community management of children on chronic invasive ventilation | An interdisciplinary working group was set up involving experts, who developed four questions to guide clinical practice with these children. | - When faced with a child on chronic mechanical ventilation who is transitioning to the community, teams should adopt a collaborative approach to care management, clarifying discharge criteria and providing appropriate training. The availability of cardiorespiratory monitoring equipment in the home environment, as well as equipment for dealing with emergencies requiring an airway approach, has also been identified as facilitating this transition.  - The realisation of these guidelines will improve the quality of life of these children when they are in home care within their community. |
| Stochitoiu & Vadeboncoeur [127] | 2020  Canada | Primary - Quantitative | To evaluate the use of the interRAI Family Carer Needs Assessment by carers of children receiving PPC; to assess the carer's ability to recognise the needs of the instrument. | A study with a prospective design, with the application of an evaluation questionnaire to a convenience sample of around 30 carers of children in PPC. | - In PPC, the existence of unmet parental needs is often perceived, such as the lack of resources for the caregiver's rest, the adaptation of the home for a child receiving palliative care and the poor identification of needs related to the caregiver.  - The interRAI Family Carer Needs Assessment is an instrument designed to identify the unmet needs of carers of people who require medical support. Used mostly in an adult context, the tool is effective in identifying the needs of family carers of these children. |
| Svavarsdottir et al. [83] | 2020  Iceland | Primary - Quantitative | To evaluate the benefit of two sessions of therapeutic dialogue oriented towards family strengths implemented by nurses for mothers of children and adolescents recently diagnosed with a CI | The study had a quasi-experimental design, including pre- and post-test comparisons through the application of a questionnaire before and after the implementation of the two interventions. | - Therapeutic dialogue focused on family strengths is an intervention that promotes a therapeutic relationship between nurses and the parents of a sick child. This methodology of care allows for in-depth knowledge of the family's constitution and functioning, which is then used to identify strengths, resilience and important resources to facilitate the experience of these episodes of illness.  - Cognitive and emotional support from the family, the development of positive beliefs about the illness, the possibility of emotional expression and understanding the impact of the illness on family life are factors that facilitate this intervention, and positive response patterns associated with it have been identified, particularly in terms of adjusting to a new reality, developing resilience and improving family functioning. |
| Ulisses et al. [141] | 2021  Brazil | Primary - Qualitative | Understanding nursing actions for the dehospitalisation of mechanically ventilated children | Interviews were carried out with 15 nursing professionals from a paediatric de-hospitalisation unit. The results were subjected to content analysis and interpreted using Roy's Adaptation Model. | - The process of de-hospitalising a ventilator-dependent child involves an advanced and integrated approach, starting with the recognition of the problem, followed by a socio-familial assessment, the identification of the child's and parents' needs and the planning of care accordingly.  - Faced with a defined plan for this process, the ability to adapt and the feeling of autonomy and security are patterns of positive response in the family. |
| Verberne et al. [84] | 2017  The Netherlands | Primary - Qualitative | Learn about the dynamics of how parents care for a child with LLI from their lived experience. | Interviews were carried out with 42 parents of 24 children receiving PPC, the results of which were subjected to thematic analysis. | - Faced with the demands of these conditions, the parents of these children identify that integrated care, from the most basic to the most complex, that is organised, safe and equipped with the appropriate treatment makes it easier to deal with these situations.  - Adopting an integrated approach and sound decision-making generates positive response patterns in the parents - particularly in terms of recognising effective parenting in the face of complexity - in the child - symptomatic and disease control - and in the family - a reconfigured sense of life and family balance. |
| Verberne et al. [124] | 2017  The Netherlands | Primary - Qualitative | To understand the support provided by a PPC team from the parents' perspective. | Interviews with 42 parents of children under the intervention of a multidisciplinary paediatric palliative care team, with thematic analysis of the data. | - The existence of specialised PPC teams that extend to the home is valued by parents as an important intervention for the child, as is the assignment of a case manager.  - In these teams, attributes such as continuity and coordination of care, ease of contact and the sensitive and trusting attitude of their members are mentioned as facilitating the transition to home.  - These teams play an important role not only in managing the child's needs, but also in raising awareness of life beyond the carer's role - responsibilities towards other family members and the importance of psychosocial support. |
| Weaver & Wratchford [112] | 2017  USA | Narrative Literature Review | Recognise the influence of religion, family and culture on the spiritual development of adolescents with palliative needs | Search in PubMed and EBSCOHost. Nine articles were included in the review. | - Considering the multidimensional impact of palliative needs on adolescents, spirituality is identified as a protective dimension of their experience.  - The inclusion of spirituality as a focus of attention in PPC is therefore essential not only from a diagnostic perspective, but also from an intervention perspective. There is evidence of positive response patterns associated with spirituality, including family adjustment, adherence to therapy and quality of life in adolescents with these conditions. |
| Wells et al. [85] | 2017  USA | Primary - Mixed Methods | Evaluate the usefulness of post-discharge home visits to identify and address health problems resulting from recent hospitalisation for CMCs | Implementation of home visits to 36 children, which were evaluated using quantitative (visit time and rate of visits with evidence of problems) and qualitative (telephone interview) measures. | - Home visits in the post-discharge period were identified as a viable approach for the safe transition of care between hospital and home for these children, proving useful in identifying problems arising from hospitalisation and facilitating adaptation to the home context. The nurse's performance, and specifically the time made available to explain the dynamics associated with care to parents, is a factor valued by parents during these visits.  - Carrying out these visits makes it possible to achieve positive response patterns, such as satisfaction with care, symptom control and a reduction in the number of visits to the emergency room and hospitalisations. |
| Wightman et al. [86] | 2019  USA | Primary - Qualitative | Describe the experience of parents as carers of children on chronic dialysis for end-stage renal failure | Semi-structured interviews with 35 parents of children on peritoneal dialysis or haemodialysis, the content of which was subjected to thematic analysis. | - In the context of end-stage renal disease under haemodialysis or peritoneal dialysis, various factors coexist that make the experience of carers complex, related to the unexpected diagnosis, the practically total responsibility for managing the disease, the high stress this entails and the associated stigma. The mourning of the ideal child is also a source of negative feelings.  - In this situation, the parents' personal and professional goals are often sacrificed, as there are no resources for the carer to rest, which also limits socialising relationships. The financial impact is one of the biggest complicating factors. Negative responses are generated as a result of these factors, such as overload, social isolation, reduced family functioning and marital distancing.  - Faced with the experience of parenting a child with chronic kidney failure, adaptive behaviours (mindfulness, exercise, relaxation), spirituality, trust in the healthcare team, and the development of compassionate and supportive relationships with other parents of children on dialysis and with nurses are seen as protective factors. These have the potential to generate positive responses, focused on acceptance of the child's state of health, socio-emotional adjustment and pragmatic adaptation, as well as personal growth. |
| Yazdani, Chartrand & Stacey [104] | 2022  Canada | Primary - Qualitative | To explore parents' and healthcare professionals' perspectives on parental experiences of decision-making between acute and palliative interventions throughout the child's LLI trajectory | Semi-structured interviews with 6 parents and 6 nurses, the results of which were subjected to thematic analysis. | - Decision-making about a child's LLI is a complex process, with significant emotional burden, high stress and uncertainty. These situations often generate negative response patterns, such as decisional conflict.  - Factors that facilitate decision-making are identified, such as confidence in beliefs, the perception of control over parental wishes, shared decision-making with professionals and the peace of mind associated with the existence of a professional support network (emotional and informative) and parental support (sharing with other parents). This enhances the recognition of an adequate parental role and the perception of self-efficacy. |
| Young [138] | 2023  USA | Case Study / Project | Describe how the Indiana Complex Care Coordination Collaborative works | Support programme for transitional nursing care for children with CCC. | - The Indiana Complex Care Coordination Collaborative is a collaborative coordination programme for children with CCC in a community setting. By training nurses as care coordinators, these professionals are responsible for managing cases not only clinically, but also socially and in the community.  - The action of these nurses is based on three pillars: organisation, with a particular focus on time management; communication, where each family is guided towards the development of a care plan with shared objectives; and collaboration, liaising with other professionals according to perceived needs. This programme generates positive results, associated with an improvement in the lives of children and their families, directly motivated by the presence of the care coordinator. |
| Zhang et al. [117] | 2014  China | Primary - Quantitative | Comparing the management of chronic illness in children among different Chinese families, identifying predictors of their management style | Questionnaire applied to a sample of 387 family carers. Statistical analysis was carried out after data collection. | - Various family management styles have been described, with difficult, insecure and disorganised management styles being seen as hindering the experience of chronic illness, stimulating the development of negative response patterns focused on the difficulty of managing CCC and compromising family functioning.  - The styles that are seen as most facilitating in the management of the illness are the realised, collaborative and competent management style, giving rise to more positive response patterns, such as effective management of the illness, parental reciprocity and the child's functionality. |
