# Supplementary material for: Health-illness transition processes in children with complex chronic conditions and their parents: a scoping review
Source: BMC Pediatr. 2024 Jul 11;24:446. doi: 10.1186/s12887-024-04919-4 (PMC11238377; doi:10.1186/s12887-024-04919-4)
Supplement: Supplementary file 5 — Supplementary Material 5. [file 12887_2024_4919_MOESM5_ESM.docx]

**Additional File 5**

Synthesis of results under *Pager Framework*

**Additional File 5**

**Synthesis of results under *Pager Framework***

| **Patterns** | **Advances** | **Gaps** | **Evidence for practice** | **Recommendations for research** |
| --- | --- | --- | --- | --- |
| **Nature of the transitions** | Understanding how to manage the multidimensionality of care and the psychosocial needs of children with CCC and their parents in hospital and community settings | Lack of studies on developmental transitions in children with CCC  Defining the profile and prevalence of needs of children with CCC and their families | Development of a care process focused beyond the health-disease situation, with interventions related to the psychosocial aspect | National and international prevalence studies on children with CCC  Studies focusing on the development of children with CCC |
| **Facilitating conditionalisms** | Valuing positive communication, empowering relationships, and family support as the main facilitating factors | Few studies on the role of therapeutic play and spirituality  Communication challenges based on multiculturalism | Focus on the communication approach and socio-family assessment as important elements of the care plan, valuing the importance of partnership with parents | Studies on the impact of programs related to therapeutic play and spirituality in mediating the transitional process |
| **Inhibiting conditionalisms** | Clinical complexity, ineffective coordination between teams, prejudices about children with CCC and economic difficulties as the main barriers | Few studies on children's adaptation to CCC  Particularization of policies that hinder the management of CCC | Operationalization of the professionals' approach focused on their inter-collaboration and articulation  Social intervention to minimize economic difficulties | Qualitative studies on the child's perception of adaptation to CCC and social difficulties  Publications on the importance of adjusting policies to the needs of these children and parents |
| **Health interventions** | Implementation of PPC, together with empowerment, articulation with the community and advanced care planning considered to be the main interventions | Few studies on interventions aimed at children with CCC, although they are mentioned as facilitators  Technology's contribution to CCC intervention | Implementing PPC from diagnosis onwards  Integrative approach to advanced care planning | Studies on the contribution of digital health to intervention for children with CCC  Descriptive studies / experience reports on the implementation of PPC in this context |
| **Patterns of response** | Effective self-management, well-being and quality of life as the main positive response patterns  Social isolation and decreased quality of life as the main negative response patterns, especially in the diagnostic phase | Few studies on the relationship between the personal and professional lives of parents who take on the role of caregiver  The child's reported perspective on the CCC situation | Encourage positive response patterns early on, while monitoring quality of life and any negative effects (stress, overload, etc.) | Studies focused on the impact of CCC on parents' professional lives  Studies aimed at understanding children's perspectives on CCC in their daily lives |
